# Genome-Wide Chromatin Structure Changes During Caste Differentiation in Honeybees

**DOI:** 10.3390/cells15201899

**Published:** 2026-10-19

**Authors:** Xinru Zhang, Hanbing Lu, Xinying Qu, Lingjun Xin, Rongshen Wang, Chongbo Liang, Xiaopeng Liu, Xiao Chen

**Affiliations:** 1 State Key Laboratory of Resource Insects, Institute of Apicultural Research, Chinese Academy of Agricultural Sciences, Beijing 100193, China; 202330112016@bua.edu.cn (X.Z.); 821012530463@caas.cn (H.L.);; 2 College of Bioscience and Resource Environment, Beijing University of Agriculture, Beijing 102206, China; 3 Shijiazhuang Animal Disease Prevention and Control Center, Shijiazhuang 050026, China; 4 Beijing Municipal Landscape and Greening Industry Promotion Center (Beijing Edible Forest Products Quality and Safety Center), Beijing 101118, China; 5 Gansu Provincial Apiculture Technology Extension Station, Tianshui 741020, China

**Keywords:** *Apis mellifera*, Caste differentiation, RNA-seq, WGBS, Hi-C, *Mrjp1*

## Abstract

Three-dimensional (3D) chromatin architecture is an important transcription regulator which is linked to gene expression regulation and correct developmental programs, yet little is known that the 3D chromatin architecture within honeybees and how it influences gene expression and methylation in caste differentiation. Here, we report that larvae at 96 h after hatching (L5) contain the highest number of known caste differentiation genes, and that constitutes the critical window determining the final phenotypic outcomes. We also show that DNA methylation pattern is conserved and gene body methylation is thought to contribute to phenotypic plasticity. Then, we determine the 3D organization of honey bee chromosomes at 5 kb resolution. Although almost 87% regions remained in the same compartments, TAD boundary insulation increased during larvae development, especially in worker larvae at 96 h after hatching (WL5). The insulator protein, CP190, CTCF-L and vtd/Rad21 appear to be indispensable in defining these topologically associating domain boundaries. Furthermore, *Mrjp1*, a member of the MRJP/yellow family, is also involved in regulating larval development. These results provide key insights into caste differentiation and establish a fundamental dataset for the future in-depth characterization of epigenetic features.

## 1. Introduction

Many organisms have the capacity to produce contrasting organismal outcomes from one genotype using intricate developmental cues [1,2]. This captivating biological phenomenon known as phenotypic plasticity is considered one of the most interesting albeit poorly understood properties of biological systems. It is found in most striking representation in eusocial insects, such as ants and honey bees.

Honey bees (*Apis mellifera*), as insect pollinators, play a crucial role in most ecosystems and strongly influence ecological relationships [3]. Honey bees are eusocial insects forming two phenotypically distinct castes: i.e., short-lived functionally sterile workers and long-lived fertile queens [4]. Despite their identical genomes, queens and workers exhibit tremendous differences in reproductive capacity, morphology, longevity, and behaviors. These differences largely depend on feeding nutrition during the early larval stage (within the first 96 h) [5]. During the initial 72 h post-hatching, both queen larvae (QL) and worker larvae (WL) were provided with a certain amount of nutritious jelly. Nonetheless, the worker jelly contains lower concentration of sugars and a few other ingredients than queen food known as royal jelly [6,7]. Larvae committed to the worker caste are then shifted to a diet comprised of nectar and pollen, whereas queen-destined larvae continue feeding exclusively on royal jelly [3]. After 96 h, brood cells are sealed, and larval feeding ceases entirely until pupation is complete. Therefore, the honey bee genome exemplifies environmentally induced phenotypic plasticity, where diet determines the emergence of discrete caste phenotypes from a single genome and represents one of the most striking examples of developmental plasticity in any phylum [3].

Various mechanisms are proposed for this phenotypic plasticity in honey bees. RNA sequencing [8] or combined with genomics and epigenomics (e.g., DNA methylation [9,10,11], histone modification [3,12,13], RNA m6A modification [14]) were implemented to unravel the intricate genetic mechanism of phenotypic plasticity in honey bees. Tian et al. found that the compartmentalization and CTCF binding peaks generally weakened upon DNA methylation dioxygenase Tet triple knock-out (Tet-TKO), which resulted in a weakened topologically associating domain (TAD) structure and depletion of long-range chromatin loops. Previous studies showed that histone post-translational modifications (including methylation and acetylation) are critical to shaping the chromatin structure and regulating gene expression [15,16,17,18]. Overall, these studies reveal distinct epigenomic characteristics are not independent layers of regulation but act cooperatively, via direct and indirect pathways, to orchestrate the hierarchical folding of chromatin. Nevertheless, because these studies mainly focus on the linear or local epigenomic changes, it remains unclear how the three-dimensional (3D) organization of the genome contributes to the initiation and progression of caste-specific phenotypic plasticity in honeybees.

Within eukaryotic cell nucleus, genomic DNA is hierarchically folded and spatially organized to form a complex 3D structure, including A/B compartments, topologically associating domains (TADs), and chromatin loops [19,20]. Chromatin conformation capture techniques, such as high-resolution chromosome conformation (Hi-C), have been developed to probe the 3D genome organization of genomes at high resolution and reveal gene regulatory mechanisms [21,22]. Nowadays, these approaches have been widely applied to multiple species, involving cancer development, spermatogenesis, folliculogenesis and silk secretion [23,24,25,26]. Although the higher-order genome folding of 3D genome architecture is conserved from insects to mammals, 3D genomics studies have uncovered that A/B compartment switching and TADs dissolution and re-establishment still occur during development [27]. Moreover, the 3D genomic organization and genome alterations can influence each other [24,28,29]. These findings implicated that 3D genome dynamic changes may represent an important but less-explored regulatory layer, particularly in honey bee caste differentiation.

Based on linear DNA sequencing technology is still difficult to clear integrate the mechanism of phenotypic plasticity in honey bees. The phenotypic plasticity is commonly coupled with genomic alterations such as gene expression changes and methylated modification. It is important to characterize the spatial disorganization of the honey bee larvae genome and determine its functional consequences. In this study, we investigate the transcriptomic dynamics and DNA methylation of QL and WL at three key developmental stage (48 h, 72 h and 96 h after hatching) and generate higher resolution chromatin contact maps for QL and WL using in situ Hi-C sequencing. We also reported changes in gene expression associated with the disorganized 3D genome, with the aim of better understanding the molecular mechanism of phenotypic plasticity in honey bees.

## 2. Materials and Methods

### 2.1. Sampling

All samples were obtained from *Apis mellifera ligustica* honeybee colonies. The queen was artificially inseminated with semen from a single drone, and then lays eggs normally. To obtain WL of known age, frames containing eggs were removed from healthy hives and placed as soon as possible in an incubator at 35 °C and ∼80% humidity, in a warm room (at least 30 °C), according to Wojciechowski et al.’s methods [3]. The locations of individual newly hatched larvae were marked on a sheet, and after that the frames were immediately returned to their original hives. The exact time was recorded. After hours, the frames were transferred back to a warm room and the larvae were collected at 48 h (L3), 72 h (L4) and 96 h (L5), respectively. To obtain QL of known age, standard queen raising techniques were used [3,30].

### 2.2. RNA-Seq Experiments and Analysis

Total mRNA with polyA tail was extracted from three biological replicates at each of the three stages (48 h, 72 h, and 96 h after hatching) in both QL and WL, and reverse-transcribed to cDNA for sequencing. The RNA-seq was performed by the Smart-Seq2 method [31]. Qualified libraries were then loaded on Illumina Hiseq platform for PE150 sequencing. Sequencing libraries were created according to the manufacturer’s protocol. Sequencing data were aligned to the *Apis mellifera* reference genome (4.5) using HISAT2 (v2.1.0) [32]. String Tied (v1.3.2.d) was then used to assemble transcripts from the mapped reads [33]. FPKM for each gene were calculated. Briefly, the DEGs were identified using DESeq2 (v1.20.0) [34](https://bioconductor.org/packages/release/bioc/html/DESeq2.html, accessed on 4 April 2025; local dispersion fit) based on the raw count data, following the standard DESeq2 workflow. Prior to hypothesis testing, the “independent filtering” function within DESeq2 was employed to automatically filter out genes with low expression levels. Normalization was performed using size factors method within DESeq2 to correct for differences in sequencing depth between samples. Trended dispersion estimation was performed to improve the accuracy of differential testing. *p*-value used a negative binomial distribution calculation model. The effect sizes forpairwise comparisons across the six stages were reported with 95% CIs. The significant DEGs were screened with a false discovery rate (FDR) < 0.05 and |log2fold change| ≥ 1 as cutoffs.

#### 2.2.1. Identifying Stage-Specific Expression Genes Set with JS Score

To better identify stage-specific expression genes in each condition, we further calculated a tissue specificity score for each transcript using an entropy-based metric that relies on Jensen–Shannon (JS) divergence according to Cabili’s methods [35]. This specificity metric (ranging from 0 to 1) quantifies the similarity between a transcript’s expression pattern across tissues and another predefined pattern that represents the extreme case in which a transcript is expressed only in one tissue. Thus, a perfect tissue-specific pattern will be scored as JS = 1.

#### 2.2.2. K-Means Clustering with DEGs

Firstly, we calculated mean log2 FPKM value (add 0.001 to avoid zero) of each gene in each condition, respectively. Then, we used K-means R function to obtain 6 clusters with all DEGs between 15 comparable groups of pairs (iter.max = 10,000, scale by samples for each gene before run K-means). The heatmap plot was generated by pheatmap R package 1.0.13.

#### 2.2.3. GO and KEGG Functional Analysis

The GO (Gene Ontology, http://geneontology.org/) and KEGG (Kyoto Encyclopedia of Genes and Genomes, http://www.kegg.jp/) [36] enrichment of DEGs was implemented by the hyper-geometric test (Fisher’s exact test), in which *p*-value is calculated and adjusted as q-value, and data background is genes in the whole genome. Only GO and KEGG terms with q < 0.05 were considered significant.

### 2.3. WGBS Experiments and Analysis

Genomic DNA was isolated from three biological replicates in each of the six groups with the Micro Elute Genomic DNA Kit (Omega Bio-tek, Norcoss, GA, USA), according to the manufacturer’s protocol. The genomic DNA library was constructed using the Swift Bioscience Accel-NGS (next generation sequencing) Methyl-Seq DNA Library Kit. Subsequently, library quality was assessed on the Agilent 2100, and 150 bp paired-end sequencing of each sample were performed on the Illumina HiSeq 4000 platform.

The clean reads after quality control were aligned to the *Apis mellifera* reference genome (4.5) using Bismark (v0.16.3) [37]. The methylation information was extracted after filtering the duplicate reads using (bismark_methylation_extractor --no_overlap) and visualized in Integrative Genomics Viewer (IGV) [38]. Bisulfite conversion efficiency was estimated as the proportion of cytosine reads matching reference cytosine positions. The genome was partitioned into 10 kb bins for inter-sample methylation correlation analysis and 3.2 Mb bins for Circos plots. Methylated and unmethylated CpG, CHG, and CHH sites was counted for all samples. Differentially methylated regions (DMRs) between QL and WL were calculated with the R package-MethylKit 3.23 (1000-bp sliding window, 500-bp overlap, fisher’s exact test and FDR < 0.05) [39]. DMRs were identified using DSS, requiring an absolute methylation difference (ΔmCG) ≥ 0.1, *p*-value < 0.00005, and at least 3 CpG sites per region [40]. Hypergeometric test was used to calculate the enrichment scores and significant *p*-values of these DMRs in different chromatin states.

### 2.4. In Nucleus Hi-C Experiments and Analysis

The Hi-C experiment was performed from one individual at each of the three stages in QL and WL, following the in situ Hi-C protocol [41]. For each individual, two independent libraries were generated as technical replicates to assess library construction and sequencing stability. The Hi-C libraries were amplified by 8–10 cycles of PCR and sequenced in Illumina HiSeq instrument with 2 × 150 bp reads.

#### 2.4.1. Hi-C Data Processing

Mapping, filtering, correction and generation of Hi-C matrices were performed using HiC-Pro (v2.7.8) [42] (https://github.com/nservant/HiC-Pro, accessed on 24 June 2025). Briefly, the high-quality read pairs were mapped independently to the *Apis mellifera* reference genome 4.5 using Bowtie2 [43] (end-to-end algorithm and ‘‘-very-sensitive’’ option) with HiC-Pro (v2.7.8) [42] default parameters. After filtering, valid pairs were used to generate raw and ICE normalized matrices [42,44] at 5 kb, 10 kb, and 100 kb bin resolution.

#### 2.4.2. A/B Compartments Analysis

We identified A/B compartments at 20 kb resolution using the matrix2compartment module in cworld software (https://github.com/dekkerlab/cworld-dekker/, accessed on 15 June 2025) as previously described. The expected contact values were calculated by LOWESS smoothed averaging of intrachromosomal interactions. Log_2_-transformed observed/expected (O/E) ratios were generated, and Pearson correlations of bin interaction patterns were obtained for PCA. Chromatin was partitioned into two spatially distinct compartments according to the sign of the first principal component. The compartment with higher gene density was defined as compartment A, and the other as compartment B.

#### 2.4.3. TAD Analysis

TADs were identified with 10 kb resolution ICE-normalized interaction matrices using a Perl script matrix2insulation.pl (https://github.com/dekkerlab/crane-nature-2015, accessed on 23 June 2025 ) [44]. Insulation Scores (IS) [45] were calculated for each chromosome bin and valleys of IS identified TAD boundaries with --is = 20, --ids = 10, --nt = 0.1. TADs smaller than 30 kb were filtered in our analysis. TAD structure closeness was measured by calculating TAD scores (internal contacts)/(external contacts).

#### 2.4.4. Loops Analysis

The loops were called by Juicer HICCUPS [46] (https://github.com/aidenlab/juicer, accessed on 27 June 2025) using parameter setting “-r 5000 -f 0.01 --ignore_sparsity” and valid pairs. For Hi-C resolution calculation, the whole genome was divided into bins of the same size (1M, 500k, 200k, 100k, 40k, 20k, 10k, 5k, 1k), and then vaild pairs were used to stat the coverage of every bin. We sorted the bins in descending order according to the coverage of bins and stat the depth of 75th, 80th, 90th. At a specific resolution, resolution adequacy was determined when the 80th bin depth minimum reached 1000 reads. This criterion achieved 5 kb resolution, enabling loop analysis.

#### 2.4.5. Defining Chromatin States and Distinguish TAD Activities

Bam files were obtained after bowtie2 [43] alignment; we used ChromHMM [47] (http://compbio.mit.edu/ChromHMM/, accessed on 3 July 2025) software to identify 15 states. Briefly speaking, first, we ran “BinarizeBam” mode with 200 bp to obtain binary files, and used “LearnModel” model to obtain segments of whole-genome regions.

#### 2.4.6. Motif Analysis

De novo motif discovery: As TAD boundaries are relatively conservative among QL and WL, we used WL5 sample TAD borders (10 kb) as candidate sequences to identify motifs by using meme-suite [48] (http://meme-suite.org/, accessed on 8 August 2025); specifically, Meme and Dreme were used de novo to discover motif logos, and tomtom was used to annotate them. Finally, only two motif logos were obtained with default parameters named CTCF (MA0531.1) and vfl (MA1462.1), respectively. A total of 143 insect PFMs (non-redundant) motifs were download from JASPAR202060 [49] (http://jaspar.genereg.net/, accessed on 14 August 2025) as the database for annotation.

Enrichment motif analysis: MotifScan [50] (http://bioinfo.sibs.ac.cn/shaolab/MAmotif/index.php, accessed on 20 August 2025) was used to analyze enriched motifs and sites for candidate promoter regions with 1 × 10^−4^ *p*-value cutoff, where all honeybee promoter regions were regarded as the control. “genomecompile” and “motifcompile” commands were run to obtain a reference index and background motif score cutoff under different *p*-values.

### 2.5. Candidate Genes Identification

The *Mrjp1* coding sequence (1299 bp, NM_001011579.1) was synthesized and cloned into pcDNA3.1 vector. To obtain the same age larvae, the queen was caged to restrict it from laying eggs on a specific comb for 12 h. On the fourth day after queen caging, the randomly selected larvae were grafted from the brood comb into a 48-well culture plate. This time was defined as 0 h. All obtained *Apis mellifera* WLs were reared in the laboratory following the method described by Schmehl et al. [51]. Feeding was conducted with 2 μL *Mrjp1* overexpressed (1 μg/μL) or blank vector at 48th, 72nd and 96th larvae. Twenty-four hours after the third feeding, six larvae each group were collected, and six larvae of each group were left to continue growing normally until emergence. Morphometric characteristics (body weight, tergite 3 length, tergite 4 length and basitarsus length) were measured at 24 h according to Ruttner and Meixner (Table S1) [52,53]. Then, a one-way analysis of variance (ANOVA) was conducted to compare different groups.

Total RNA was extracted from larvae samples using Trizol reagent (Invitrogen, Carlsbad, CA, USA), and then reverse-transcribed to cDNA using M-MLV FIRST STRAND KIT (Invitrogen, Shanghai, China) and oligo (dT)18 primer following the supplier’s instructions. Transcript quantification was performed using SYBR Green mix (Roche Diagnostics GmbH, Roche Applied Science, Mannheim, Germany) and Roche LightCylcer 480 (Roche Diagnostics GmbH, Roche Applied Science, Mannheim, Germany) in a standard 96-well block (20 μL reaction; incubation at 95 °C for 10 min, 45 cycles of denaturation at 95 °C for10 s, annealing/extension at 60 °C for 10 s). *Mrjp1* Transcript specific primer pairs (F: 5′-CACAGCCCAAGATGGAATTT-3′; R: 5′-AAGAGGACGCCACTCTTTGA-3′) were designed with Oligo 6.0 software [54]. β-actin served as the internal reference gene, and relative expression was analyzed using the 2^−ΔΔCT^ method. ANOVA and *t*-test were conducted to analyze the RNA expression in different groups using software SPSS 22.0 [55].

## 3. Results

### 3.1. Dynamic Transcriptome Differences Between QL and WL

**Figure 1:**
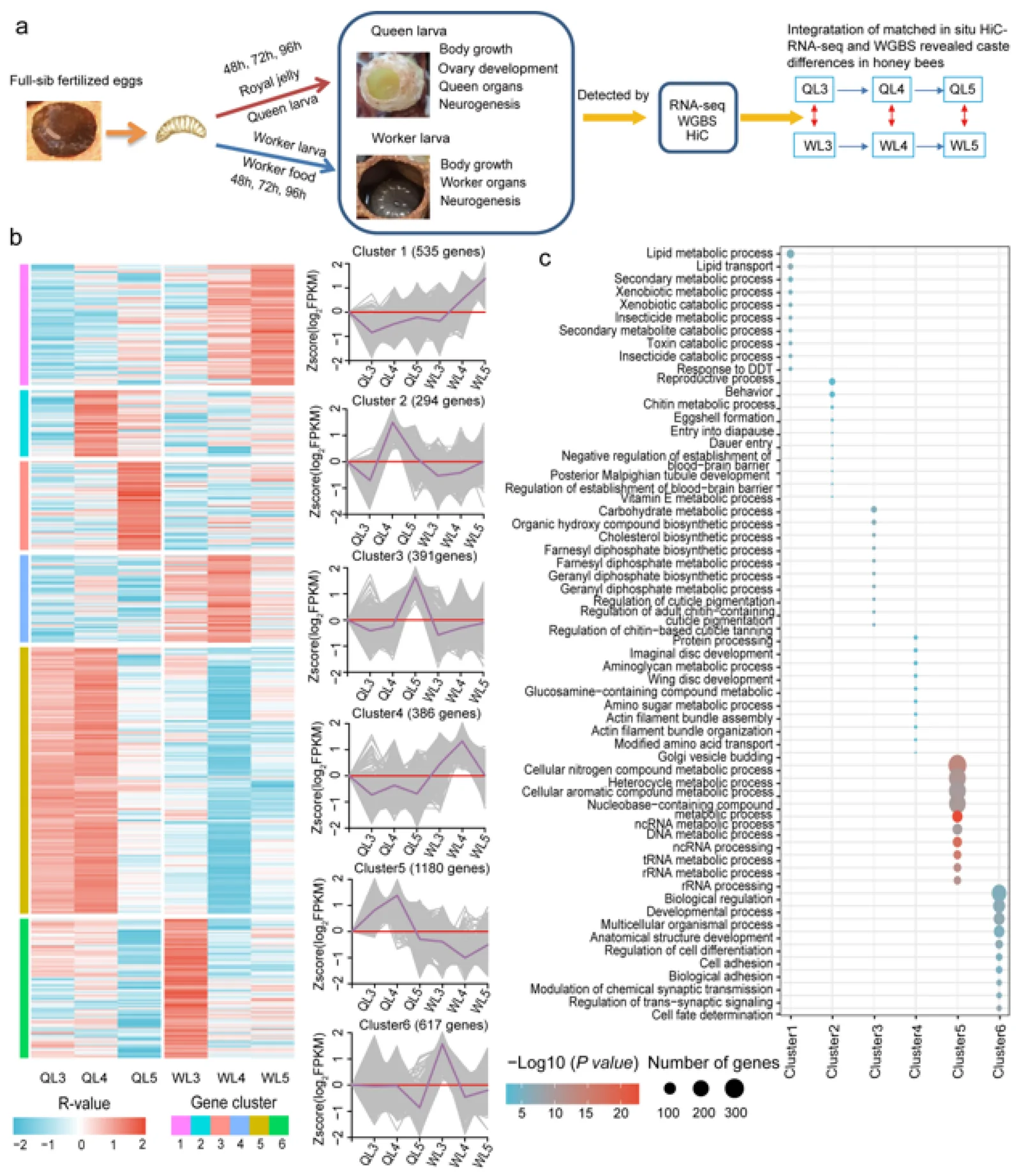
The transcriptomic profiles of L3 to L5 in honey bee caste differentiation. (**a**) Schematic representation of this study design; (**b**) expression K-means clustering profiles across six developmental stages. Left: Expression heatmap drawn using Z-score of FPKM values for each gene in the six clusters. Right: Expression trends (Z-score (log2FPKM)) of genes within each cluster at different developmental stages. The purple lines represent mean gene expression levels, and the gray lines represent gene expression levels for each gene in the relative cluster during caste differentiation; (**c**) the top ten enriched GO-BP terms for genes in each cluster.

In honey bees, female larvae are multipotent and can develop either into workers or queens with both organisms showing distinct phenotypic features. We first depicted developmental changes in the transcriptome of QL and WL at 48 h (L3), 72 h (L4) and 96 h (L5) after hatching (Figure 1a). We then generated a total of 122.89 Gb high-quality bulk RNA-seq data with three biological replicates for each group (~6.83 Gb sequences per sample) (Table S1). A total of 10,331 genes (73.32% of the annotated genes in the genome) had evident expression (fragments per kilobase million [FPKM] ≥ 0.5 at least at one instar time) during the QL and WL caste differentiation, which typically had a developmental stage-dependent pattern and is highly reproducible within biological replicates (Spearman’s r > 0.80) (Figure S1a,b and Table S2). Comparisons among developmental stages revealed that the L4 stage had the largest number of differentially expressed genes (DEGs), with 1215 genes upregulated in QL and 760 genes upregulated in WL (Figure S1c). This is consistent with the result of Zhang et al., that more DEGs were up-regulated in QL than WL [56]. Next, a functional annotation analysis was performed for the DEGs between QL and WL (Tables S3 and S4). Gene Ontology (GO) enrichment analysis suggested that these DEGs are primarily involved in cell part, cellular process and binding (Figure S1d). Overrepresented GO-BP terms were related to “estrogen biosynthetic and metabolic process”, “lipid metabolic process” and “organic acid metabolic process” (Figure S1e). Overrepresented KEGG pathways were “Steroid hormone biosynthesis” “Apoptosis” and “Glycosphingolipid biosynthesis” (Figure S1e). These results highlight the differences in biological functions occurring in honey bee caste differentiation.

Additionally, we investigated possible developmental scenarios for gene expression throughout caste differentiation in QL and WL and identified six main patterns involving 3403 genes using the maSigPro-GLM algorithm (Figure 1b and Table S5). We found that a total of 1538 genes (535, 386 and 617 genes in cluster 1, 4 and 6, respectively) were upregulated in WL. In contrast, a total of 1865 genes (294, 391 and 1180 genes in cluster 2, 3 and 5, respectively) were upregulated in QL. The genes mostly belong to cluster 5 and they show a reverse expression pattern at L4 stage, with high expression in QL4 and low expression in WL4. However, we did not find any genes known to be associated with caste differentiation in cluster 5, and most of these genes are of unknown function. Functional enrichment analysis suggested that these genes primarily involved nucleic acid metabolism and small-molecule compound metabolism, which could affect gene expression during growth and development of organisms (Figure 1c). The larvae had basically completed the differentiation of queen bee and worker bee at L5. We speculated that the L4 stage lays the foundation for L5 the final caste formation stage, and that the L4 differential genes should play an important role in caste differentiation and need to be explored more deeply in the future.

Considering the limited tissue number and the large number of low-expression genes and zeros in RNA-seq, the commonly used metrics, such as Tau, were not suitable for honey bees [57]. To confirm that over-representative functions of stage-specific genes align with specific physiological changes during caste differentiation in honey bees, we further calculated a stage specificity score for each transcript using an entropy-based metric that relies on Jensen–Shannon (JS) divergence. Based on this measure, a comprehensive analysis of differentially expressed genes and stage-specific genes (JS score > 0.3) found that QL and WL exhibit similar gene expression pattern, most stage-specific genes being highly expressed in their respective developmental stages, with only one gene down-regulated in QL4 and QL5 (Figure S2a,b and Table S6). This result showed that using the JS score method to analyze specific genes expressed in the honeybee larvae stage is reliable. Further, we performed functional annotation analysis to find specific overrepresented functional terms for stage-specific genes (Figure S2c, Tables S7 and S8). Neural development is the most significant overrepresented term at all stages of WL and in the QL3 stage. The reproduction process and tissue development were highly overrepresented at QL4. At the QL5 stage, reproductive behavior and morphogenesis were highly overrepresented. In addition, we found the most genes related to caste differentiation at the L5 stage, for example *Y-h*, *Mrjp2*, *Jhbp-1*, *Jhe*, *Mrjp7*, *Mrjp8* in WL5 and *Br-c*, *E74*, *Y-e3*, *Y-y*, *Mrjp1*, *Mrjp3* in QL5 [58]. This aligns with the finding that L5 contains the highest number of known caste differentiation genes in K-means cluster, indicating that dynamical gene expression with a developmental stage-dependent manner is related to functional divergences during caste differentiation from L3 to L5.

### 3.2. DNA Methylation Profiles of QL and WL

DNA methylation is one of the main epigenetic factors regulating gene expression in eukaryotes. To investigate the role of DNA methylation in caste differentiation in honey bees, QL and WL were isolated for whole-genome bisulfite sequencing (WGBS). After removing, bisulfite sequencing (BS-seq) yielded 336 Gb of clean data from 18 samples (three biological replicates for each of the six groups) (Table S1). To confirm the accuracy and reliability of the collected samples, we performed a correlation analysis for all samples. The results showed that the replicates from the same groups clustered relatively closely and the samples from the same stage correlated strongly (r^2^ ≥ 0.94), indicating the high repeatability of the BS-seq results (Figure S3a).

Next, the global DNA methylation status of QL and WL was investigated. The results revealed that the global methylation levels were weakly in honey bees (4.31% in QL and 4.35% in WL, Figure S3b). The classification of mC contexts (mCG, mCHH, or mCHG) for each sample showed that the proportion of mCGs was the highest, and was mainly identified in gene body regions (Figure S3c–e). This indicated that mCG is the main type of mC, and this DNA methylation pattern is conserved during caste differentiation in honey bees.

**Figure 2:**
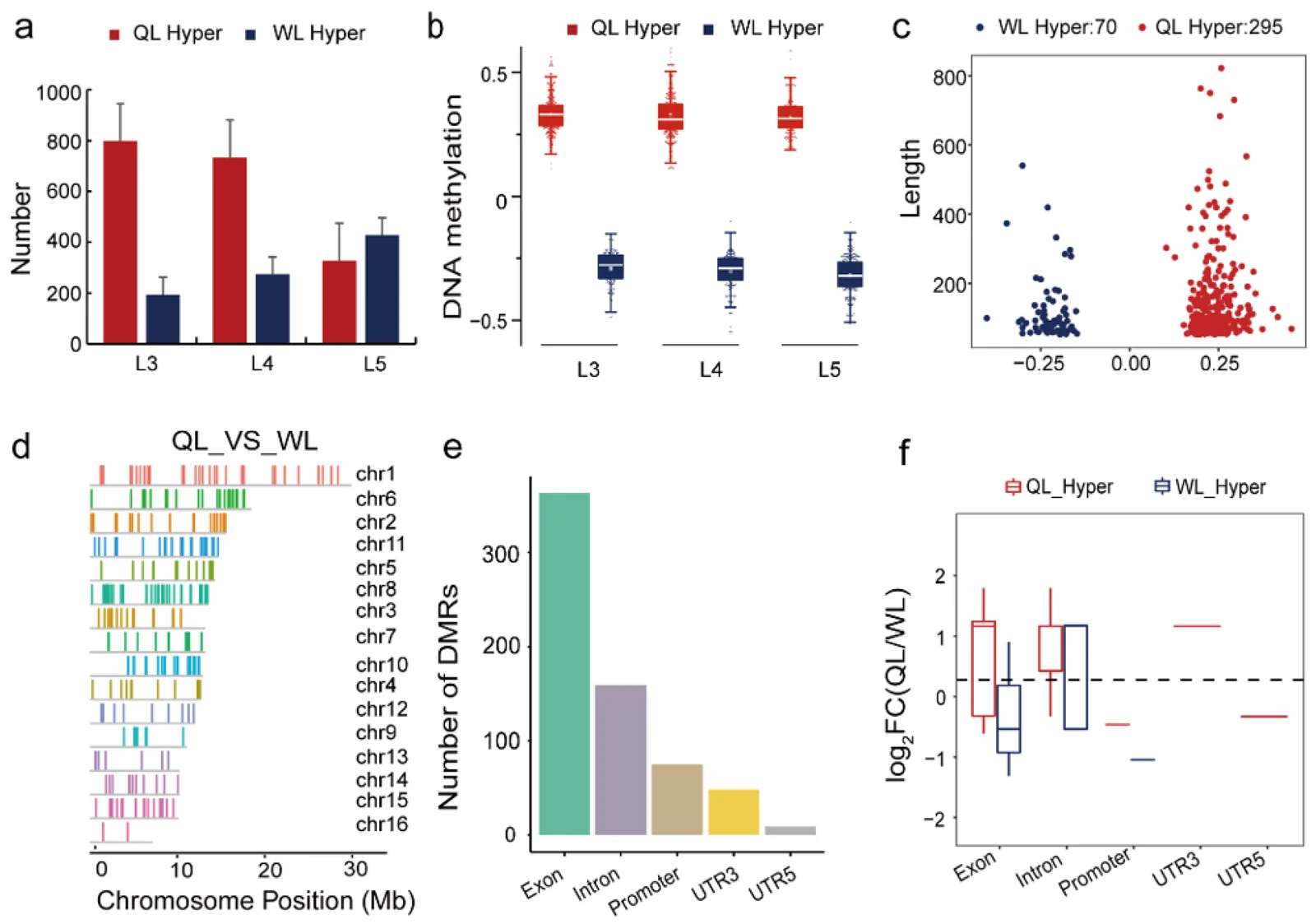
DMRs between QL and WL. (**a**) The number of DMRs from comparisons of each stage between QL and WL; number in red indicates hyper-DMRs in QL and number in blue indicates hyper-DMRs in WL. (**b**) The DNA methylation levels from comparisons of each stage between QL and WL; (**c**) the plot of specific DMRs between QL and WL; number in red indicates hyper-DMRs in QL and number in blue indicates hyper-DMRs in WL. (**d**) The distribution of specific-DMRs in chromosomes; (**e**) the number of specific DMRs in the specific genomic elements; (**f**) boxplot shows the relationship between higher expressed genes and hyper-DMRs for the specific genomic elements of QL and WL. The dashed horizontal line indicates the threshold for log2 fold change.

Differentially methylated regions (DMRs) are important markers of epigenetic changes, and are involved in the regulation of many biological processes. In this study, the number of DMRs (ΔmCG ≥ 0.1, FDR < 0.05) was 991, 1007 and 756, respectively. Compared to L3 and L4, a large number of DMRs were down-methylated in QL5 (Figure 2a,b). Then, we identified specific DMRs (only detected in one larval stage) in QL and WL to explore the transient epigenetic reprogramming events. We identified a total of 365 specific DMRs, including 295 hyper-DMRs in QL and 70 hyper-DMRs in WL (Figure 2c and Table S9). The DMRs were distributed on all chromosomes, mainly in Chr1 (Figure 2d) Among the 365 DMRs, 333 DMRs were annotated in the gene body region (Figure 2e). We also identified that the hyper-exon DMRs were associated with more highly expressed genes (Figure 2f). These findings suggested that the global methylation is low in honey bees, yet it may suggest the involvement of gene expression in honey bee caste differentiation.

### 3.3. Genome-Wide Chromatin Interaction Maps for QL and WL

**Figure 3:**
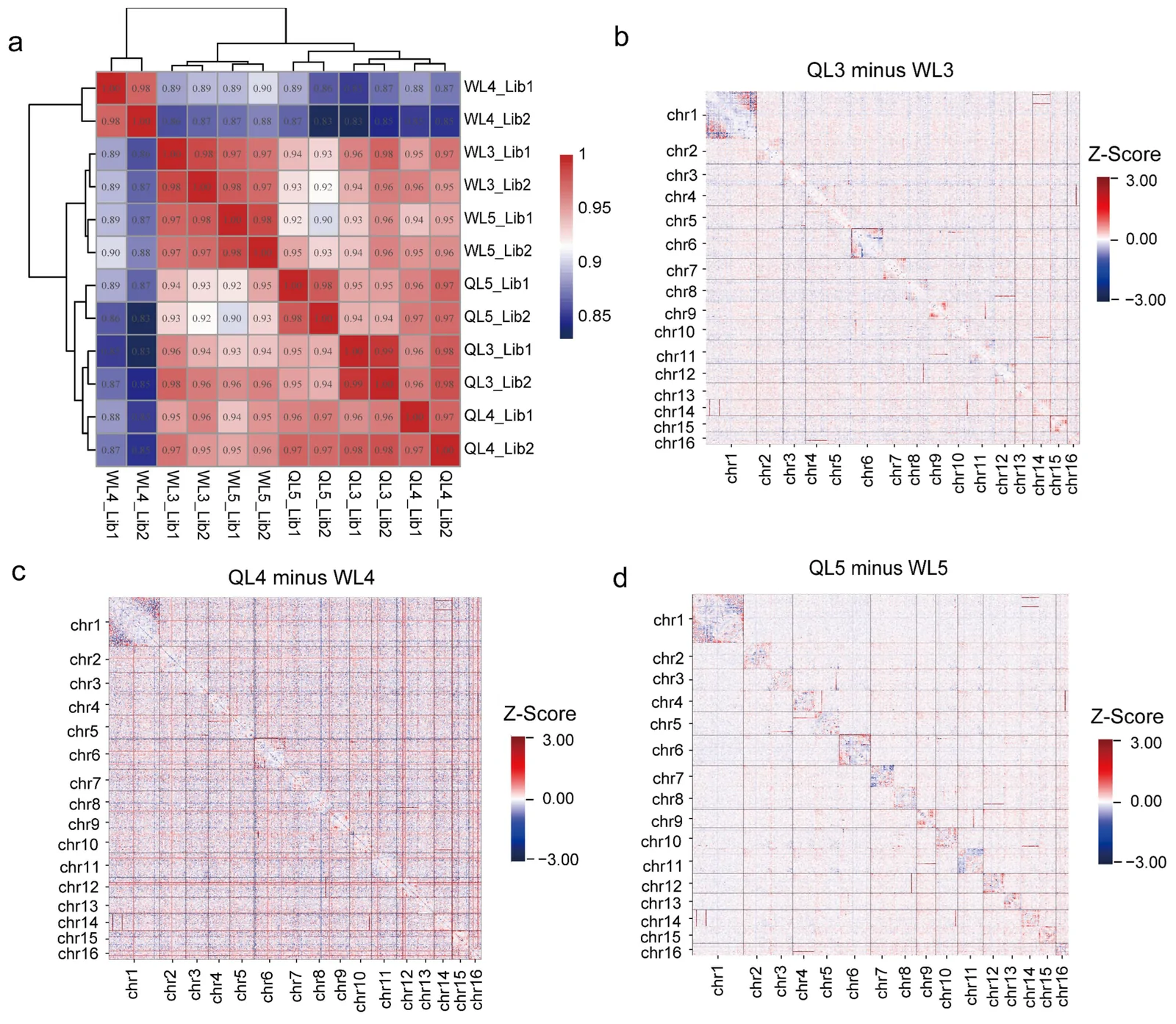
The Hi-C data with QL and WL. (**a**) The stratum-adjusted correlation coefficient heatmap plot of Hi-C libraries calculated by hicrep; (**b**–**d**) genome-wide differential chromatin interaction heatmap plots between QL and WL at L3 (**b**), L4 (**c**) and L5 (**d**) stage; shown is the 100 kb binned heatmap depicting the Z-score by using ICE normalized matrices (see Methods for Z-score difference calculation).

To study the genome-wide chromatin interactions of the phenotypic plasticity, we performed in situ Hi-C experiments of QL and WL with the restriction enzyme (MboI). The Hi-C data indicated an average depth of 5 kb. Sequencing the Hi-C library generated 5.86 billion raw reads, which yielded 5.66 billion high-quality read-pairs after all filtration steps (Figure S4a and Table S1). The stratum-adjusted correlation coefficient heatmap plot of Hi-C libraries were calculated by hicrep, and the results showed that the data sets were highly correlated (Figure 3a, Pearson’s correlation, r > 0.91). All samples showed a strong decrease interaction frequency with an increase in the genomic distance (Figure S4a). Like other species, the proportion of intra-chromosome interaction (cis-) identified in honey bees exceeds that of inter chromosome interaction (trans-), although the interaction patterns vary across different instars (Figure S4b). Additionally, we identified the resolution depth 1 kb to 1 Mb across all different stages and the intrachromosomal interaction ratio of bin resolution at 100 kb in all chromosome (Figure S4c,d). Then, we obtained chromatin interaction heatmaps at 100 kb resolution for each sample using combined data of replicates (Figure S5). After analyzing the Z-score difference in genome-wide chromosomal interaction at the same stage, we found that there are many inter-chromosomal interactions (i.e., non-diagonal regions, which occupied a larger proportion of the heatmap) in the L4 stage, whereas only Chr1 displayed pronounced differences in intra-chromosomal long-range interactions. In addition, we detected extensive differences in intra-chromosomal interactions at the L5 stage, suggesting remodeling of local chromatin folding, potentially involving TAD–internal interactions and enhancer–promoter loops (Figure 3b–d and Figure S6). In general, we propose that L5 is a key period in determining the final phenotypic outcome.

### 3.4. A/B Compartments Are Conserved During Larvae Development

**Figure 4:**
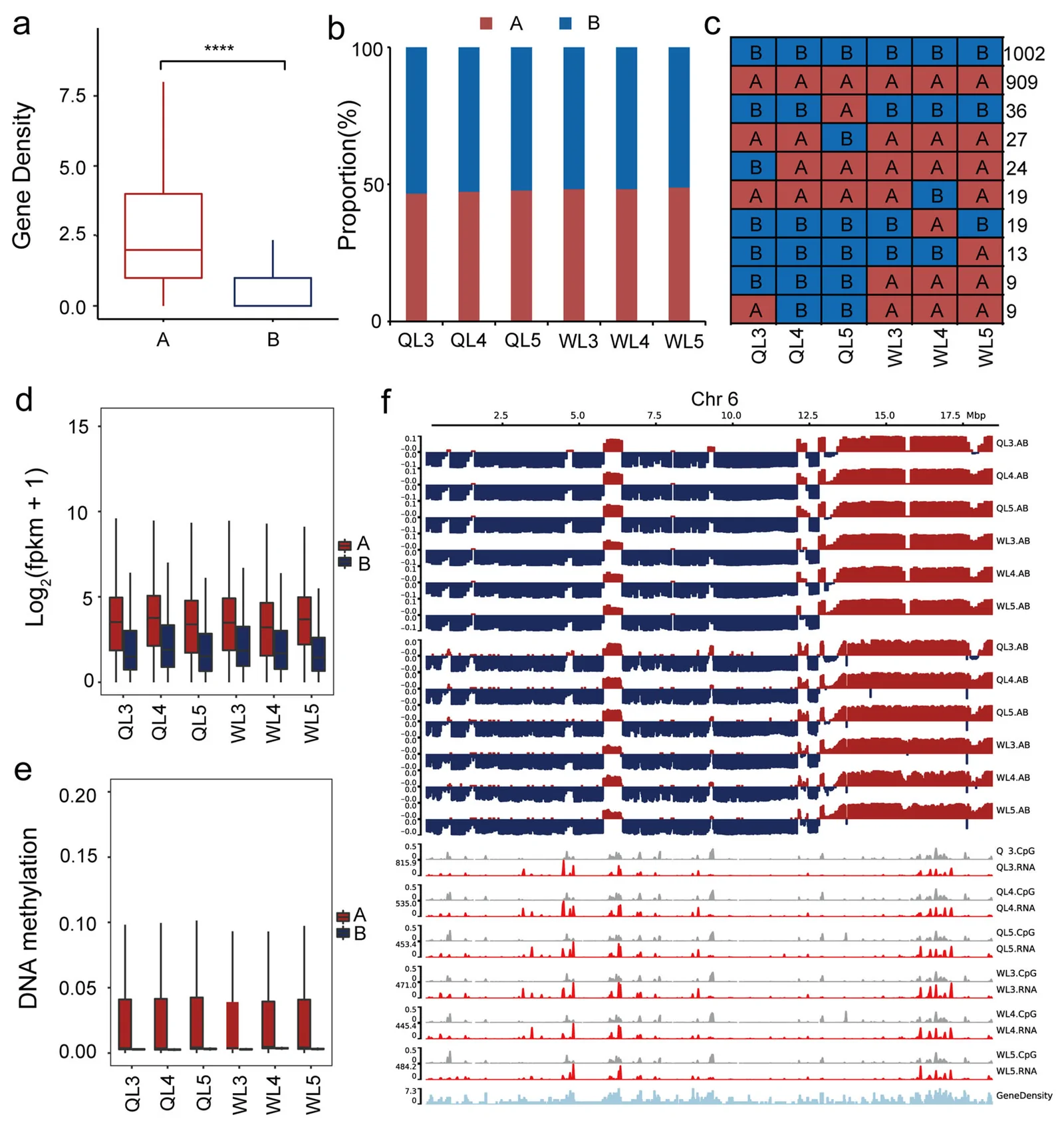
The switching of A/B compartments is associated with corresponding changes of gene expression and methylation. (**a**) Gene density in A/B compartments at 20 kb; (**b**) proportion of A/B compartments in QL and WL; (**c**) number of A/B switching compartments identified during larvae development; (**d**) gene expression (log2(FPKM + 1) in A/B compartments at 20 kb; (**e**) DNA methylation in A/B compartments at 20 kb; (**f**) gene expression and CpG methylation in A/B compartments in Chr 6. ****, *p* < 0.0001.

To elucidate the multiscale rewiring of chromatin architecture and its influence during caste differentiation, at the sub-chromosome level, we firstly characterized the compartment partitioning scenarios and examined associations between transcriptional features/methylation levels and chromatin compartment states. In honey bees, we used ICE-normalized interaction matrices at 20 kb resolution to detect chromatin compartment types and obtained 2205 chromosome bins. The genome consists of actively transcribed compartments A and inactive compartments B (Figure 4a). There are 16 chromosomes in genome of honey bees, at around 229.12 Mb [59]. Replicates of each stage shared similar A and B compartment patterns, and ~47.27% of the whole genome comprised Compartment A bins in QL and ~48.44% in WL (Figure 4b). Previous studies showed that the switching of compartments A and B between normal cells and cancer cells is associated with corresponding changes of gene expression [60]. We investigated whether this phenomenon is exhibited in honey bee caste differentiation. Finally, 909 A compartments were found associated with up-regulated gene expression and 1002 B compartments were found associated with down-regulated gene expression (Figure 4c). Most genomic regions (87%) remained in the same compartments from L3 to L5 during caste differentiation process in QL and WL. It indicated that the compartments A and B are highly conservative during the caste differentiation process. We identified a substantial level of compartmental switching in the caste differentiation process from L3 to L5. In these regions, most switching was unidirectional (74.15%): from A to B (“AAB” and “ABB”) or from B to A (“BAA” and “BBA”) (Figure 4c). We were also interested in the association between the reprogramming of these epigenetic modifications, gene expression and 3D chromatin structure. We analyzed the association between the methylated CpGs and gene expressions. We found that compartments A have higher gene expression and methylation than compartments B, for example in chromosome 6 (Figure 4d–f). These results indicated that A/B compartments are globally conserved, but local compartment switching may contribute to caste-specific gene expression and epigenetic regulation.

### 3.5. TAD Boundary Insulation Is Increased During Larvae Development, Especially in WL5

**Figure 5:**
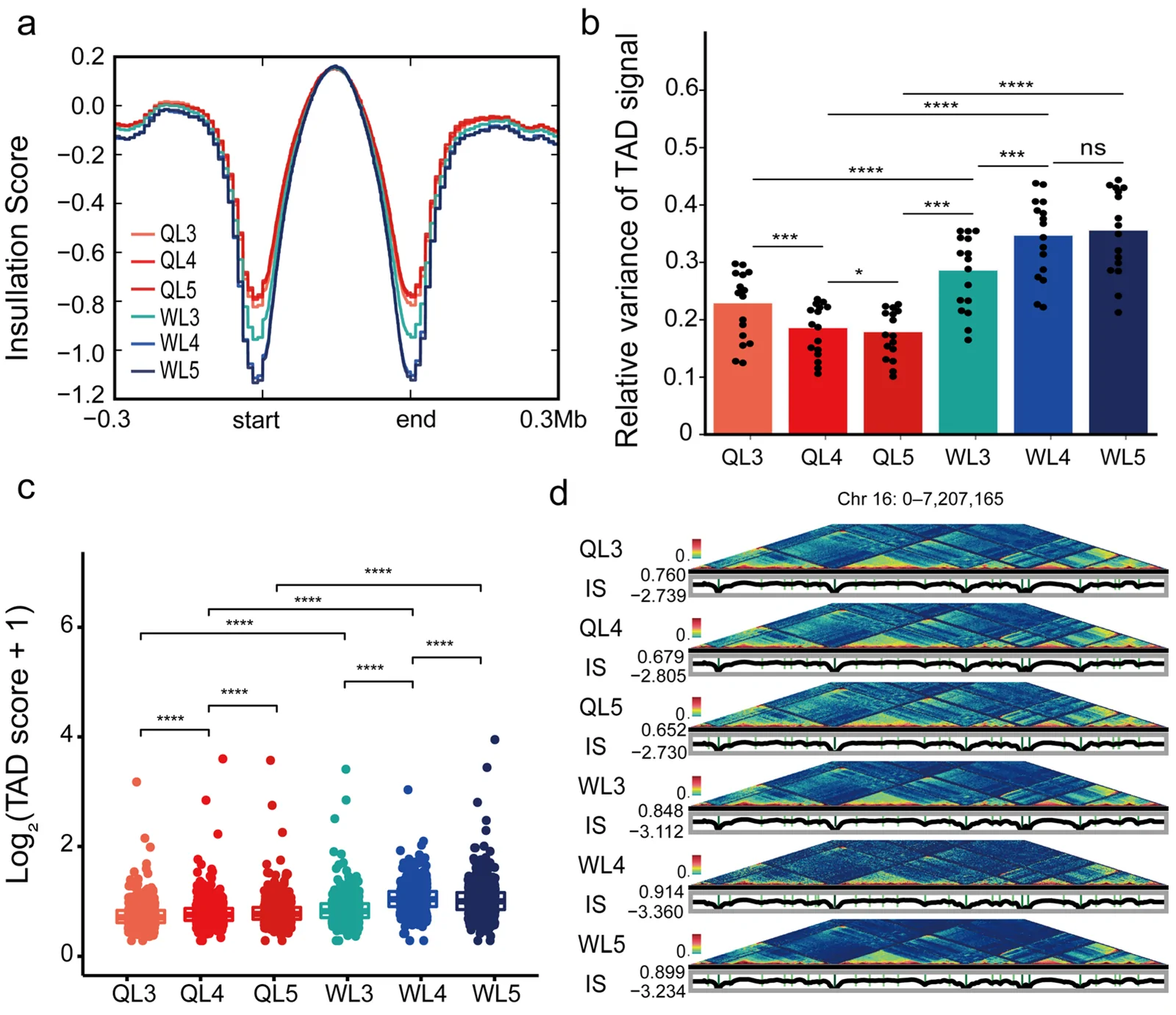
Characteristics of TADs in QL and WL. (**a**) The insulation of TADs in QL and WL; (**b**) the relative variance of TAD signal in QL and WL (Wilcoxon test pair); (**c**) TAD score boxplot using WL5 as reference (*t*-test pair); (**d**) heatmap of TADs in QL and WL in Chr16: 0–7,207,165. *, *p* < 0.05; ***, *p* < 0.001; ****, *p* < 0.0001; ns, not significant.

At the sub-megabase scale, the local chromatin architecture can be characterized by TAD. To investigate the changes in TAD in caste differentiation, we used an insulation score (IS) approach to identify TAD boundaries at 10 kb resolution [45,61]. As a result, we found that the genome was shown to consist of small contiguous TADs in honey bees, a property also observed in *Drosophila* and mammals [61]. To identify all of the sub-TADs’ borders, a high-sensitivity threshold was used. A total of 2085 TADs were subsequently detected in QL and WL, and the TAD number was the highest at L5 (Figure S7a and Table S10). The identified TADs exhibited the smallest median size in WL5 (552 kb in WL5) (Figure S7b). Furthermore, we observed a stronger TAD boundary insulation at WL than that in QL (Figure 5a). Next, we intersected their TAD boundaries to test whether the TAD boundaries were gained or lost in QL and WL. We found that the precise location of our TAD boundaries exhibited a high degree of overlap (at least 91%) in QL and WL (Figure S7c). Only a small portion of TAD boundaries were unique to their period. Analyzing the DEGs at L5-specific TAD boundaries demonstrated that these genes could be candidate genes affecting caste differentiation (Table S11). In addition, we also found that the methylated CpGs were mostly located at TAD boundaries. However, the results showed that the methylated CpGs of genes at TAD boundaries did not show significant changes. Overall, most TADs identified in QL and WL were stable during caste differentiation.

To assess the kinetics of intra-TAD interactions during the larval development, we calculated the domain score and signal (defined as the TAD compactness and reflect the self-interactive tendencies within a domain) of cTADs of each sample. Using the TAD interaction strength from the WL5 as a reference, we observed a reverse interaction trend in QL and WL, and the relative variance of TAD signal and TAD score were significantly enhanced and increased at WL5 (Figure 5b,c). For example, the TAD borders in Chr 16 (0–7,207,165) gradually became more and more clear, indicating increased spatial segregation of local chromatin when the caste differentiated (Figure 5d). This is consistent with our analysis of TAD characteristic, including the number, the length and the conservation. This further confirms that the strongest chromatin interaction occurs at this stage (Figure 3d).

### 3.6. Genes Expression and Methylation at TAD Boundary

**Figure 6:**
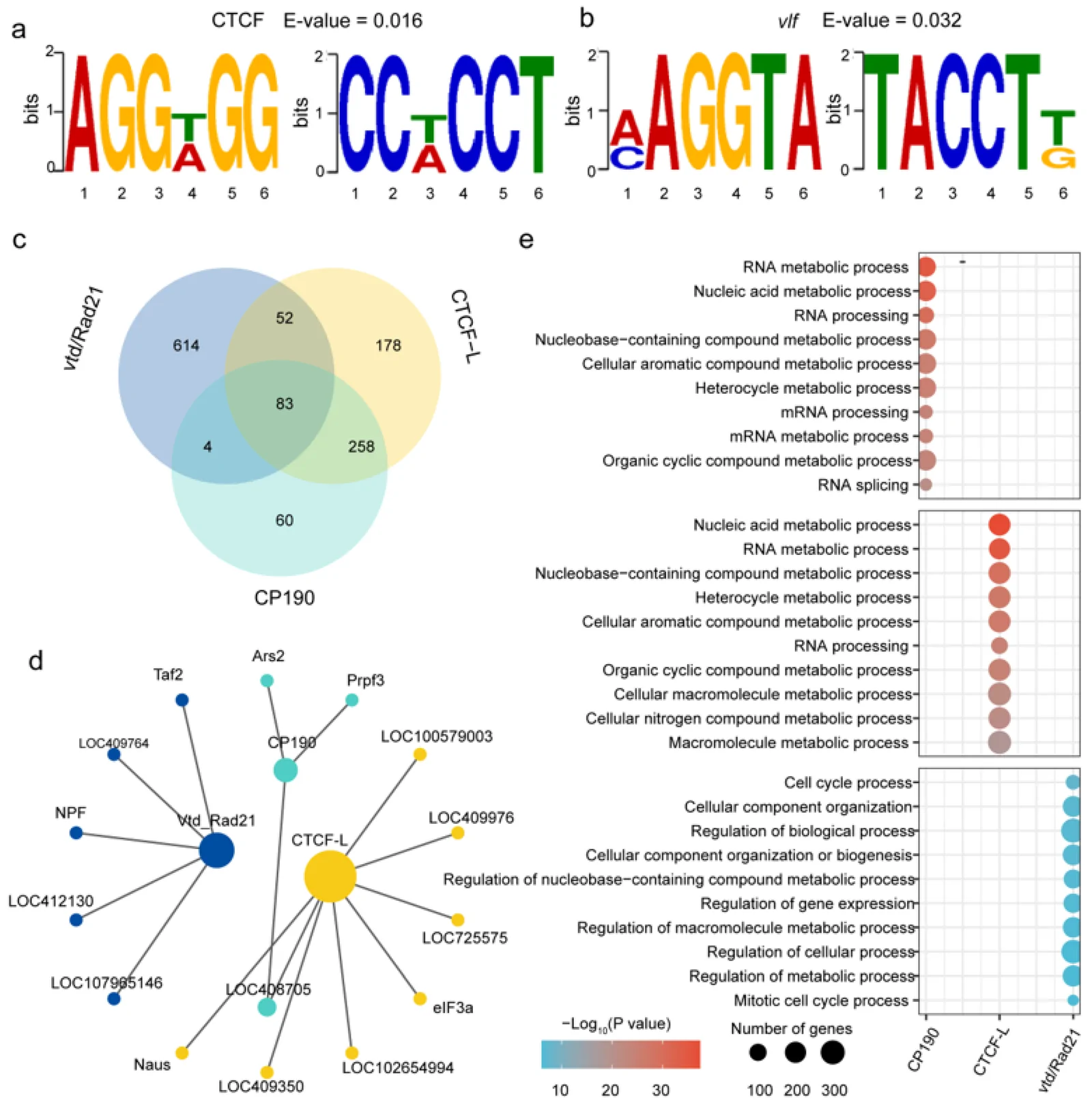
Motif predict at TAD boundary. (**a**,**b**) The most significantly enriched motifs identified within honeybees using DREME; the left shows the forward strand, and the right shows the reverse complementary strand. (**a**): CTCF. (**b**): *Vfl*. (**c**) Venn diagram of key genes involved in these three motifs with absolute Spearman correlation coefficient > 0.7; (**d**) interaction relationships between TAD boundary-associated genes and other genes (absolute Spearman correlation coefficient > 0.9). (**e**) The top 10 enriched GO-BP terms for CP190, CFTF-L, and vtd/Rad21.

To further explore what precisely regulates the insulation strength of TAD boundaries in honey bees, we used Meme&Dreme to perform Motif de novo analysis of the TAD boundary sequences, and then motif name annotation on the identified motifs using tomtom. Then, we successfully detected the top two motif sequences with dreme: CTCF and vfl (top 1 and 2, respectively) (Figure 6a). By homology alignment, we found that CP190, CTCF-L, and vtd/Rad21 were highly homologous to the motif sequence, which may play an analogous role in honey bees as of in *Drosophila*. For the purpose of determining the intergenic interaction relationships between genes and identifying the genes with central roles, we performed co-expression analysis of TAD-associated key genes at absolute Spearman correlation coefficient value (abs_SCC) > 0.7. A total of 83 genes exhibited strong expression correlations with all of the three predicted motifs (Figure 6b). We also observed that there were 15 genes strongly linked with the motifs (abs_SCC > 0.9), which may play an important role in 3D genome organization by adjusting the strength of chromatin insulation at TAD boundaries (Figure 6c and Table S12). Function enrichment analysis indicated that the top 10 GO-BP of CP190 and CTCF-L are mostly involved in RNA processing and RNA metabolism process, while vtd/Rad21 is mainly involved in the regulation of biological and cellular processes (Figure 6d). These results indicated that CTCF and vlf sites may play an important role in the formation of TAD boundaries in honey bees.

### 3.7. Candidate Genes Identification

**Figure 7:**
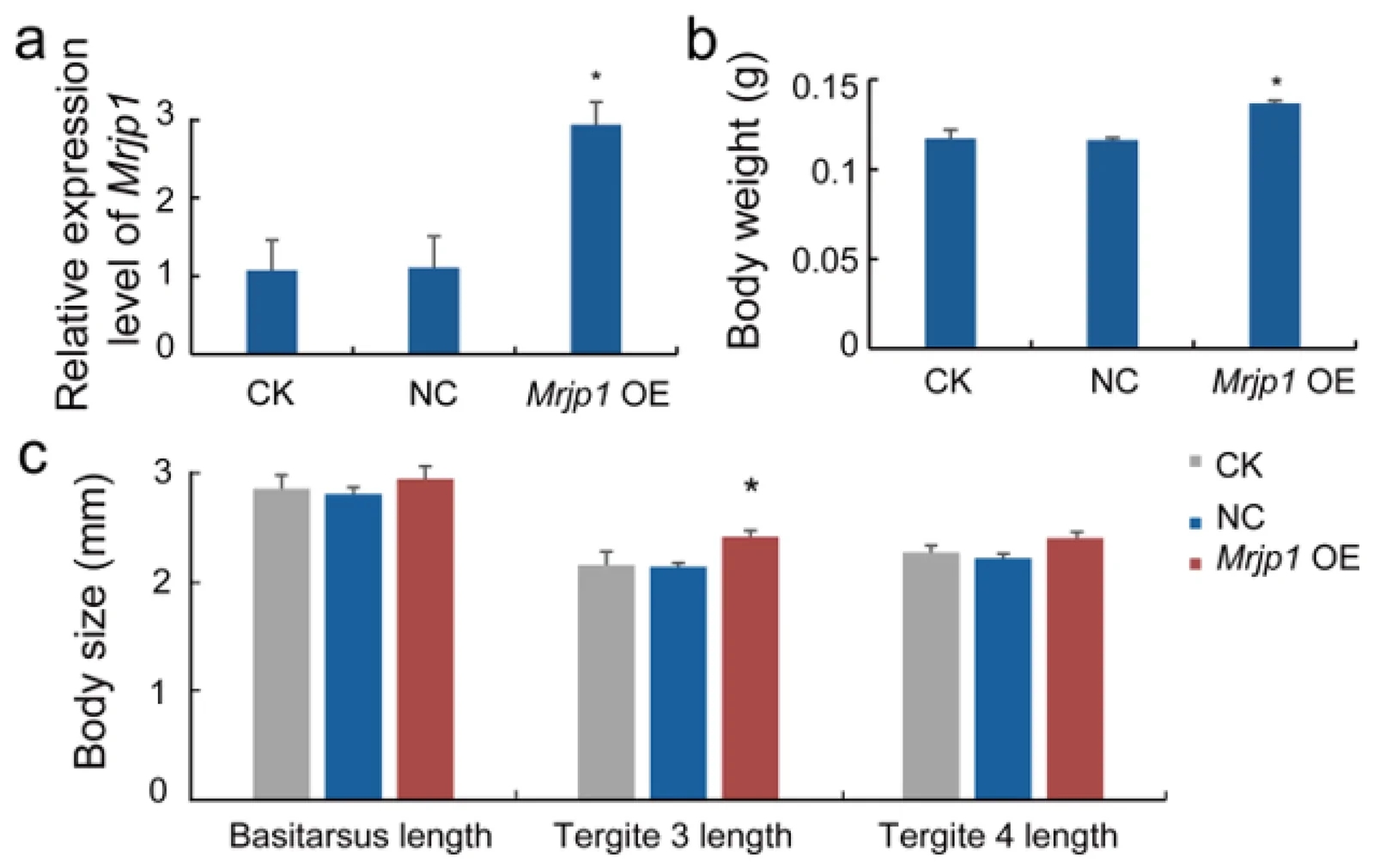
Overexpression of *Mrjp1*. (**a**) Overexpression of *Mrjp1* in WL; (**b**) the effect of *Mrjp1* overexpression on workers’ body weight; (**c**) the effect of *Mrjp1* overexpression on workers’ body size. *, *p* < 0.05.

Royal jelly (RJ) is the exclusive dietary requirement of honey bee queens and has been reported to play a critical role in caste differentiation [62,63]. The MRJP family represents the main characteristic proteins, in which MRJP1 is the most abundant and upregulated in QL [64]. The TAD results showed that the MRJP family is located in one TAD (Chr 11: 2,540,000–2,700,000 (Table S13). As mentioned above, *Mrjp1* was also located in a L5-specific TAD boundary and upregulated in QL (Table S11). Here, we overexpressed *Mrjp1* in worker bees to verify its effect on larvae development. The RT-qPCR confirmed that the overexpression of *Mrjp1* effectively increased its expression in WL. Facilitated by a vector, the overexpression (OE) of *Mrjp1* effectively increased its expression (294% compared with control group, *p* < 0.01) and led to a significant increase in emergence weight 17% and tergite 3 length 12% (*p* < 0.05) (Figure 7). Thus, these results provide evidence that the expression of *Mrjp1* has a positive effect on larvae development.

## 4. Discussion

Sequencing technology based on linear DNA sequence still does not clearly illustrate the mechanism of phenotypic plasticity in honey bees. Therefore, we assume that the genome 3D structure changes during honeybee caste differentiation may lead to the destruction of the original TAD and changes in gene interactions. This would further affect the regulation of genes and pathways associated with phenotypic plasticity. In this study, we comprehensively analyzed RNA-seq, WGBS and Hi-C with higher resolution across a key time course of honey bees in caste differentiation. Here, we confirmed that all these three omics were involved in caste differentiation. Our results showed that the L4 stage (76 h after hatching) served as the preparation period for caste differentiation, during which the chromatin started to undergo significant changes along with notable differences in gene expression and methylation. These up-regulated DEGs and stage-specific genes identified in QL4 were mainly involved in ncRNA metabolic process, providing the material and energy foundation for cell proliferation and tissue differentiation in queen larvae, especially the rapid development of their ovaries [65,66,67]. After that, we detected many genes know to be related to caste differentiation, perhaps suggesting that the caste-specific development at L5 stage is already irreversible [14,68,69,70].

In addition, we found that the global methylation levels in honey bee genomes are sparse and mostly restricted to gene bodies, which commonly occurs in social insects [70,71,72]. A large number of DMRs were down-methylated at the QL5 stage, while these were up-methylated in WL5. In 2012, Foret et al. also observed greater methylation in 96 h-old worker larval heads [10]. Compared with a previously published meDIP-seq methylation pattern, our results showed a similar trend after the fourth day: DNA methylation of QL was decreased, while that of WL continued to increase [72]. This provides support again to the notion that larvae 96 h after hatching (L5) were at a critical juncture, and WLs older than 4 days were not able to switch to becoming queens.

Interestingly, with the larval development, the TAD borders became gradually defined, sharpest at WL5. The 3D structure was extremely loose at the initial stage and lacked classical structural features, such as compartment A/B and TAD [73]. With embryonic development, the genome 3D structure and high-level structure of chromosomes gradually matured and became established [74]. This indicates that the 3D genome structure is gradually established and matured along with larvae development in honey bees. In humans, mice, fruit flies and other species, TADs exhibit higher intra-domain interaction frequency than inter-domain interactions, even in nearby areas [75,76]. We believe that TAD divides the chromosomes into independent small units to ensure spatial isolation, even if TADs are very close to each other on the linearized chromosomes. As universal structural features and fundamental units within the genome, TADs provide a physical platform for gene expression. The dynamic changes of TADs ensure precision of transcriptional dynamics and the reliability of complex patterning processes [24]. Workers are responsible for various and detailed tasks, including nurturing, guarding, gathering, etc. Indeed, we observed increased TAD signal, TAD score and insulation score in WL compared to that in QL, which is consistent with the worker bee biological characteristics. Boundaries are thought to act as insulators to preclude inappropriate enhancer–promoter interactions with neighboring genes or regulatory elements [77]. The compact TAD architecture and well-defined TAD boundaries broadly maintain regulatory specificity by preventing interference between neighboring TADs [24]. Our results suggested that combinatorial effects and insulating capacity of TAD boundaries could be an important mechanism shaping gene expression patterns and enabling the fine-scale partitioning of gene functions.

TADs provide a suitable microenvironment for fine gene expression regulation [74]. This is helpful in identify candidate genes for caste differentiation in honey bees. The MRJP/yellow family is currently recognized as playing a key role in the differentiation of honey bees. Our results indicated that overexpression of *Mrjp1* can increase body-weight- and body-size-related characteristics, whereas the interference effect of *Mrjp1* is not satisfactory, which may be attributed to compensatory intake from its presence in the fed diet [64]. Our TAD results showed that the MRJP/yellow family is located in the same TAD (Chr11: 2,540,000–2,700,000). There are 12 genes located in this TAD. The TAD scores of the 12 genes are all the highest at L4, indicating that these genes interact more, and more closely, during the larvae development. Of them, nine genes are members of the MRJP/yellow family. The overexpression of *Mrjp1* can promote larvae development and have a positive effect on workers’ body size. In this TAD, the other three genes are L-xylulose reductase (gene id: 413567) and two unknown genes. Notably, the expression level of L-xylulose reductase in honey bee larvae is significantly higher in WL than in QL, and hundreds of times higher than that of MRJPs, particularly in L4 and L5. L-xylulose reductase converts l-xylulose into xylitol, and reduces various α-dicarbonyl compounds, thus performing a particular role in carbohydrate metabolism [78,79]. Previous studies have shown that L-xylulose reductase can help maintain stable blood sugar levels and prevents cell damage caused by hyperglycemia [80,81]. We deduced that the expression difference of L-xylulose reductase between QL and WL may stem from nutrient-mediated regulatory mechanisms during critical late-stage larval development, which could play a regulatory role in caste differentiation.

Juvenile hormone (JH), a class of structurally unique sesquiterpenoid hormones in honey bees, plays crucial roles in regulating growth and development [82], labor division [83,84], lifespan [85], and caste differentiation [68,86]. At the critical window of caste differentiation, the JH titer threshold determines the developmental trajectory of the larvae (development into a queen or worker) [68,86]. Juvenile hormone esterase (*Jhe*, gene id: 406066), located in TAD Chr 3: 8,520,000–8,720,000, is the primary JH-specific degradation enzyme regulating JH titers [87,88]. Genes within this TAD may interact with JH and JHE, and collectively affect the development of honey bee larvae. Another gene in this TAD, *Titin* (gene id: 409981), contributes to sarcomere elasticity and extensibility, especially responses to changes in muscle length [89]. Another study by the authors explored the regulatory relationship among JH, *Jhe* and *Titin*, as well as their functions in honeybee larval development [90]. The results revealed that inhibiting *Jhe* expression in workers’ larvae led to a significant accumulation of JH titer during the larval stage (*p* < 0.01) and a marked downregulation of *Titin* expression (*p* < 0.05). Direct inhibition of *Titin* expression led to an increase in multiple body size traits in adult workers, including fore wing, femur and tibia length (*p* < 0.01). The results indicate that JH, *Jhe* and *Titin* interact with each other and jointly regulate the development of worker larvae [90].

At the honey bee TAD borders, we obtained the significant enrichment pairs of insulator proteins, CTCF-L and vfl. This pattern is analogous to the CTCF–cohesin enrichment at mammalian TAD boundaries and BEAF-32/CP190 or Chromator in *D. melanogaster*. Insulator proteins are known to colocalize with numerous TAD boundaries across species [61,91,92,93]. Nevertheless, there exists a notable mechanistic discrepancy: TAD borders in mammals are occupied by the CTCF/cohesin insulating complexes, whereas such complexes are absent from *D. melanogaster* TAD boundaries. This implies that the border-associated elements may not be conserved among metazoans. The insulator proteins located at TAD borders are known to be critical for TAD establishment [93,94,95,96]. In *Drosophila*, transcription has been hypothesized to serve as a primary driver of TAD architecture, though the involvement of genomic sequences bound by architectural proteins (APs) remains debated [61,97,98]. In 2020, Rodrigo et al. provided compelling evidence that discrete genomic regions bound by APs and RNA Polymerase II contribute to TAD partitioning and transcriptional regulation in *Drosophila* [98]. However, whether these proteins or their homologs play a similar function in honey bees remains uncharacterized and warrants further studies.

## 5. Conclusions

In summary, our study presents an atlas of chromatin interactions in honey bee during larvae development at 5 kb resolution. We demonstrated that higher-order chromatin architectural changes, particularly the maturation of TAD boundaries and compartmentalization, facilitate the concomitant transcriptional activities and guarantee the precision of caste differentiation. Additionally, expression changes in key functional genes such as MRJP1 and Jhe-associated genes could influence larvae development. These results imply that the very different epigenomic profiles of the worker and queen bees are not pre-established and can diverge by taking progressively different developmental pathways. It will be an interesting challenge in the future to explore the functions of genes unknown to this study and how they influence 3D genome architectural dynamics in honey bees throughout caste development, as well as analyze various epigenetic data to better understand regulatory mechanisms in phenotypic plasticity.

## Data Availability

All essential codes used for analysis are available at GitHub (https://github.com/ChengLiLab/myeloma, accessed on 16 January 2025). The Hi-C data of queen and worker larvae (QL and WL) generated in this study have been deposited in the Gene Expression Omnibus (GEO) database under accession code GSE306160. The RNA-seq and BS-seq data generated in this study have been deposited in the GEO database under accession code GSE306161 and GSE306158 via NCBI.

## Supplementary Materials

The following supporting information can be downloaded at: https://www.mdpi.com/article/10.3390/cells15201899/s1, Figure S1: Specific expression genes of QL and WL in developmental stage; Figure S2: Stage-specific expression genes of QL and WL in developmental stage; Figure S3: DNA methylation landscape in several developmental stage; Figure S4: The characteristics of Hi-C data set in QL and WL; Figure S5: The whole genome interactions in QL and WL; Figure S6: The differential whole genome interactions between QL and WL; Figure S7. Characteristics of TADs in QL and WL; Table S1: Sequencing data summary; Figure S8. Characteristics of TADs in QL and WL; Table S2: The FPKM expression matrix of 10,331 DEGs during QL and WL caste differentiation; Table S3: The top 20 GO-BP terms enriched for DEGs identified during QL and WL caste differentiation; Table S4 The top 20 KEGG terms enriched for DEGs identified during QL and WL caste differentiation; Table S5: Gene clusters derived from heatmap analysis, representing six main expression patterns across 3403 gene; Table S6: Six main classes of stage-specific genes during QL and WL caste differentiation; Table S7: The enriched GO-BP and KEGG terms for genes belonging to the JS class module in QL; Table S8: The enriched GO-BP and KEGG terms for genes belonging to the JS class module in WL; Table S9: The differentially methylated regions detected between QL and WL; Table S10: The genomic locations of TADs in QL and WL called at 10 kb resolution; Table S11: Total of 37 significant DEGs at L5 specific TAD boundaries; Table S12: TAD boundary key genes and their associated co-expressed genes filtered by absolute SCC(abs_SCC_0.7); Table S13: Annotated gene list within each TAD region, including TAD score, gene symbol and FPKM expression values.

## References

[1] Agrawal A.A. (2001). Phenotypic plasticity in the interactions and evolution of species. Science.

[2] Evans J.D., Wheeler D.E. (2001). Gene expression and the evolution of insect polyphenisms. Bioessays.

[3] Wojciechowski M., Lowe R., Maleszka J., Conn D., Maleszka R., Hurd P.J. (2018). Phenotypically distinct female castes in honey bees are defined by alternative chromatin states during larval development. Genome Res..

[4] Page R.E., Peng C.Y. (2001). Aging and development in social insects with emphasis on the honey bee, *Apis mellifera* L.. Exp. Gerontol..

[5] Kamakura M. (2011). Royalactin induces queen differentiation in honeybees. Nature.

[6] Wang Y., Ma L., Zhang W., Cui X., Wang H., Xu B. (2016). Comparison of the nutrient composition of royal jelly and worker jelly of honey bees (*Apis mellifera*). Apidologie.

[7] Maleszka R. (2018). Beyond Royalactin and a master inducer explanation of phenotypic plasticity in honey bees. Commun. Biol..

[8] Zhang W., Wang L., Zhao Y., Wang Y., Chen C., Hu Y., Zhu Y., Sun H., Cheng Y., Sun Q. (2022). Single-cell transcriptomic analysis of honeybee brains identifies vitellogenin as caste differentiation-related factor. iScience.

[9] Kucharski R., Maleszka J., Foret S., Maleszka R. (2008). Nutritional Control of Reproductive Status in Honeybees via DNA Methylation. Science.

[10] Foret S., Kucharski R., Pellegrini M., Feng S., Jacobsen S.E., Robinson G.E., Maleszka R. (2012). DNA methylation dynamics, metabolic fluxes, gene splicing, and alternative phenotypes in honey bees. Proc. Natl. Acad. Sci. USA.

[11] Lyko F., Foret S., Kucharski R., Wolf S., Falckenhayn C., Maleszka R. (2010). The honey bee epigenomes: Differential methylation of brain DNA in queens and workers. PLoS Biol..

[12] Zhang Y., Li Z., He X., Wang Z., Zeng Z. (2023). H3K4me1 modification functions in caste differentiation in honey bees. Int. J. Mol. Sci..

[13] Spannhoff A., Kim Y.K., Raynal N.J., Gharibyan V., Su M.B., Zhou Y.Y., Li J., Castellano S., Sbardella G., Issa J.P. (2011). Histone deacetylase inhibitor activity in royal jelly might facilitate caste switching in bees. EMBO Rep..

[14] Wang M., Xiao Y., Li Y., Wang X., Qi S., Wang Y., Zhao L., Wang K., Peng W., Luo G.-Z. (2021). RNA m^6^A modification functions in larval development and caste differentiation in honeybee (*Apis mellifera*). Cell Rep..

[15] Li W.S., Carter L.M., Almassalha L.M., Gong R., Pujadas-Liwag E.M., Kuo T., MacQuarrie K.L., Carignano M., Dunton C., Dravid V. (2025). Mature chromatin packing domains persist after RAD21 depletion in 3D. Sci. Adv..

[16] Zheng L., Wang W. (2022). Regulation associated modules reflect 3D genome modularity associated with chromatin activity. Nat. Commun..

[17] Millán-Zambrano G., Burton A., Bannister A.J., Schneider R. (2022). Histone post-translational modifications—Cause and consequence of genome function. Nat. Rev. Genet..

[18] Lawrence M., Daujat S., Schneider R. (2016). Lateral thinking: How histone modifications regulate gene expression. Trends Genet..

[19] Li D., He M., Tang Q., Tian S., Zhang J., Li Y., Wang D., Jin L., Ning C., Zhu W. (2022). Comparative 3D genome architecture in vertebrates. BMC Biol..

[20] Wang S., Luo Z., Liu W., Hu T., Zhao Z., Rosenfeld M.G., Song X. (2023). The 3D genome and its impacts on human health and disease. Life Med..

[21] Lieberman-Aiden E., van Berkum N.L., Williams L., Imakaev M., Ragoczy T., Telling A., Amit I., Lajoie B.R., Sabo P.J., Dorschner M.O. (2009). Comprehensive mapping of long-range interactions reveals folding principles of the human genome. Science.

[22] Fullwood M.J., Liu M.H., Pan Y.F., Liu J., Xu H., Mohamed Y.B., Orlov Y.L., Velkov S., Ho A., Mei P.H. (2009). An oestrogen-receptor-α-bound human chromatin interactome. Nature.

[23] Kloetgen A., Thandapani P., Ntziachristos P., Ghebrechristos Y., Nomikou S., Lazaris C., Chen X., Hu H., Bakogianni S., Wang J. (2020). Three-dimensional chromatin landscapes in T cell acute lymphoblastic leukemia. Nat. Genet..

[24] Batut P.J., Bing X.Y., Sisco Z., Raimundo J., Levo M., Levine M.S. (2022). Genome organization controls transcriptional dynamics during development. Science.

[25] Wang Y., Wang H., Zhang Y., Du Z., Si W., Fan S., Qin D., Wang M., Duan Y., Li L. (2019). Reprogramming of meiotic chromatin architecture during spermatogenesis. Mol. Cell.

[26] Tong Z. (2023). Comparative Study on 3D Genome Structure of Lepidoptera.

[27] Che Y., Bush S.J., Lin H., Li M., Yang X., Xie Q., Liu Y., Meng D., Ye K. (2026). The evolution of high-order genome architecture revealed from 1,000 species. Cell.

[28] Spielmann M., Lupiáñez D.G., Mundlos S. (2018). Structural variation in the 3D genome. Nat. Rev. Genet..

[29] Ding T., Fu S., Zhang X., Yang F., Zhang J., Xu H., Yang J., Chen C., Shi Y., Bai Y. (2024). Inter3D: Capture of TAD reorganization endows variant patterns of gene transcription. Genom. Proteom. Bioinf..

[30] Evans J.D., Schwarz R.S., Chen Y.P., Budge G., Cornman R.S., De la Rua P., de Miranda J.R., Foret S., Foster L., Gauthier L. (2013). Standard methods for molecular research in *Apis mellifera*. J. Apic. Res..

[31] Picelli S., Björklund Å.K., Faridani O.R., Sagasser S., Winberg G., Sandberg R. (2013). Smart-seq2 for sensitive full-length transcriptome profiling in single cells. Nat. Methods.

[32] Kim D., Paggi J.M., Park C., Bennett C., Salzberg S.L. (2019). Graph-based genome alignment and genotyping with HISAT2 and HISAT-genotype. Nat. Biotechnol..

[33] Kovaka S., Zimin A.V., Pertea G.M., Razaghi R., Salzberg S.L., Pertea M. (2019). Transcriptome assembly from long-read RNA-seq alignments with StringTie2. Genome Biol..

[34] Love M.I., Huber W., Anders S. (2014). Moderated estimation of fold change and dispersion for RNA-seq data with DESeq2. Genome Biol..

[35] Cabili M.N., Trapnell C., Goff L., Koziol M., Tazon-Vega B., Regev A., Rinn J.L. (2011). Integrative annotation of human large intergenic noncoding RNAs reveals global properties and specific subclasses. Genes Dev..

[36] Kanehisa M., Goto S. (2000). KEGG: Kyoto Encyclopedia of Genes and Genomes. Nucleic Acids. Res..

[37] Krueger F., Andrews S.R. (2011). Bismark: A flexible aligner and methylation caller for Bisulfite-Seq applications. Bioinformatics.

[38] Thorvaldsdóttir H., Robinson J.T., Mesirov J.P. (2013). Integrative Genomics Viewer (IGV): High-performance genomics data visualization and exploration. Brief. Bioinf..

[39] Akalin A., Kormaksson M., Li S., Garrett-Bakelman F.E., Figueroa M.E., Melnick A., Mason C.E. (2012). methylKit: A comprehensive R package for the analysis of genome-wide DNA methylation profiles. Genome Biol..

[40] Park Y., Wu H. (2016). Differential methylation analysis for BS-seq data under general experimental design. Bioinformatics.

[41] Rao S.S., Huntley M.H., Durand N.C., Stamenova E.K., Bochkov I.D., Robinson J.T., Sanborn A.L., Machol I., Omer A.D., Lander E.S. (2014). A 3D map of the human genome at kilobase resolution reveals principles of chromatin looping. Cell.

[42] Servant N., Varoquaux N., Lajoie B.R., Viara E., Chen C.-J., Vert J.-P., Heard E., Dekker J., Barillot E. (2015). HiC-Pro: An optimized and flexible pipeline for Hi-C data processing. Genome Biol..

[43] Langmead B., Salzberg S.L. (2012). Fast gapped-read alignment with Bowtie 2. Nat. Methods.

[44] Imakaev M., Fudenberg G., McCord R.P., Naumova N., Goloborodko A., Lajoie B.R., Dekker J., Mirny L.A. (2012). Iterative correction of Hi-C data reveals hallmarks of chromosome organization. Nat. Methods.

[45] Crane E., Bian Q., McCord R.P., Lajoie B.R., Wheeler B.S., Ralston E.J., Uzawa S., Dekker J., Meyer B.J. (2015). Condensin-driven remodelling of X chromosome topology during dosage compensation. Nature.

[46] Durand N.C., Shamim M.S., Machol I., Rao S.S.P., Huntley M.H., Lander E.S., Aiden E.L. (2016). Juicer provides a One-Click System for analyzing loop-resolution Hi-C experiments. Cell Syst..

[47] Ernst J., Kellis M. (2017). Chromatin-state discovery and genome annotation with ChromHMM. Nat. Protoc..

[48] Bailey T.L., Johnson J., Grant C.E., Noble W.S. (2015). The MEME suite. Nucleic Acids. Res..

[49] Fornes O., Castro-Mondragon J.A., Khan A., van der Lee R., Zhang X., Richmond P.A., Modi B.P., Correard S., Gheorghe M., Baranašić D. (2020). JASPAR 2020: Update of the open-access database of transcription factor binding profiles. Nucleic Acids. Res..

[50] Sun H., Wang J., Gong Z., Yao J., Wang Y., Xu J., Yuan G.-c., Zhang Y., Shao Z. (2018). Quantitative integration of epigenomic variation and transcription factor binding using MAmotif toolkit identifies an important role of IRF2 as transcription activator at gene promoters. Cell Discov..

[51] Schmehl D.R., Tomé H.V.V., Mortensen A.N., Martins G.F., Ellis J.D. (2016). Protocol for the in vitro rearing of honey bee (*Apis mellifera* L.) workers. J. Apic. Res..

[52] Ruttner F. (1988). Biogeography and Taxonomy of Honeybees.

[53] Meixner M.D., Pinto M.A., Bouga M., Kryger P., Ivanova E., Fuchs S. (2013). Standard methods for characterising subspecies and ecotypes of *Apis mellifera*. J. Apic. Res..

[54] Rychlik W., Rhoads R.E. (1989). A computer program for choosing optimal oligonucleotides for filter hybridization, sequencing and in vitro amplification of DNA. Nucleic Acids. Res..

[55] Pallant J. (2013). SPSS Survival Manual: A step by step guide to data analysis using IBM SPSS for Windows. Aust. N. Z. J. Public Health.

[56] Zhang Y., He X.J., Barron A.B., Li Z., Jin M.J., Wang Z.L., Huang Q., Zhang L.Z., Wu X.B., Yan W.Y. (2023). The diverging epigenomic landscapes of honeybee queens and workers revealed by multiomic sequencing. Insect Biochem. Mol. Biol..

[57] Kryuchkova-Mostacci N., Robinson-Rechavi M. (2017). A benchmark of gene expression tissue-specificity metrics. Brief. Bioinf..

[58] Pandey A., Bloch G. (2015). Juvenile hormone and ecdysteroids as major regulators of brain and behavior in bees. Curr. Opin. Insect Sci..

[59] Cao L., Zhao X., Chen Y., Sun C. (2021). Chromosome-scale genome assembly of the high royal jelly-producing honeybees. Sci. Data.

[60] Gong H., Yang Y., Zhang S., Li M., Zhang X. (2021). Application of Hi-C and other omics data analysis in human cancer and cell differentiation research. Comput. Struct. Biotechnol. J..

[61] Wang Q., Sun Q., Czajkowsky D.M., Shao Z. (2018). Sub-kb Hi-C in D. melanogaster reveals conserved characteristics of TADs between insect and mammalian cells. Nat. Commun..

[62] Buttstedt A., Mureşan C.I., Lilie H., Hause G., Ihling C.H., Schulze S.-H., Pietzsch M., Moritz R.F.A. (2018). How honeybees defy gravity with royal jelly to raise queens. Curr. Biol..

[63] Buttstedt A., Ihling C.H., Pietzsch M., Moritz R.F.A. (2016). Royalactin is not a royal making of a queen. Nature.

[64] Simuth J. (2001). Some properties of the main protein of honeybee (*Apis mellifera*) royal jelly. Apidologie.

[65] He X.J., Barron A.B., Yang L., Chen H., He Y.Z., Zhang L.Z., Huang Q., Wang Z.L., Wu X.B., Yan W.Y. (2022). Extent and complexity of RNA processing in honey bee queen and worker caste development. iScience.

[66] Pan Y.-H., Shan L., Zhang Y.-Y., Yang Z.-H., Zhang Y., Cao S.-M., Liu X.-Q., Zhang J., Yang L., Chen L.-L. (2025). Pre-rRNA spatial distribution and functional organization of the nucleolus. Nature.

[67] Corona M., Libbrecht R., Wheeler D.E. (2016). Molecular mechanisms of phenotypic plasticity in social insects. Curr. Opin. Insect Sci..

[68] Barchuk A.R., Cristino A.S., Kucharski R., Costa L.F., Simões Z.L.P., Maleszka R. (2007). Molecular determinants of caste differentiation in the highly eusocial honeybee *Apis mellifera*. BMC Dev. Biol..

[69] Hartfelder K., Tiberio G.J., Lago D.C., Dallacqua R.P., Bitondi M.M.G. (2018). The ovary and its genes—Developmental processes underlying the establishment and function of a highly divergent reproductive system in the female castes of the honey bee, Apis mellifera. Apidologie.

[70] Bonasio R., Li Q., Lian J., Mutti N.S., Jin L., Zhao H., Zhang P., Wen P., Xiang H., Ding Y. (2012). Genome-wide and caste-specific DNA methylomes of the ants *Camponotus floridanus* and *Harpegnathos saltator*. Curr. Biol..

[71] Terrapon N., Li C., Robertson H.M., Ji L., Meng X., Booth W., Chen Z., Childers C.P., Glastad K.M., Gokhale K. (2014). Molecular traces of alternative social organization in a termite genome. Nat. Commun..

[72] Shi Y.Y., Yan W.Y., Huang Z.Y., Wang Z.L., Wu X.B., Zeng Z.J. (2013). Genomewide analysis indicates that queen larvae have lower methylation levels in the honey bee (*Apis mellifera*). Naturwissenschaften.

[73] Hug C.B., Grimaldi A.G., Kruse K., Vaquerizas J.M. (2017). Chromatin architecture emerges during zygotic genome activation independent of transcription. Cell.

[74] Dryden N.H., Broome L.R., Dudbridge F., Johnson N., Orr N., Schoenfelder S., Nagano T., Andrews S., Wingett S., Kozarewa I. (2014). Unbiased analysis of potential targets of breast cancer susceptibility loci by Capture Hi-C. Genome Res..

[75] Lukyanchikova V., Nuriddinov M., Belokopytova P., Taskina A., Liang J., Reijnders M.J.M.F., Ruzzante L., Feron R., Waterhouse R.M., Wu Y. (2022). Anopheles mosquitoes reveal new principles of 3D genome organization in insects. Nat. Commun..

[76] Despang A., Schöpflin R., Franke M., Ali S., Jerković I., Paliou C., Chan W.-L., Timmermann B., Wittler L., Vingron M. (2019). Functional dissection of the Sox9–Kcnj2 locus identifies nonessential and instructive roles of TAD architecture. Nat. Genet..

[77] Tendolkar A., Mazo-Vargas A., Livraghi L., Hanly J.J., Van Horne K.C., Gilbert L.E., Martin A. (2024). Cis-regulatory modes of Ultrabithorax inactivation in butterfly forewings. eLife.

[78] Son L.T., Ko K.-M., Cho J.H., Singaravelu G., Chatterjee I., Choi T.-W., Song H.-O., Yu J.-R., Park B.-J., Lee S.-K. (2011). DHS-21, a dicarbonyl/l-xylulose reductase (DCXR) ortholog, regulates longevity and reproduction in *Caenorhabditis elegans*. FEBS Lett..

[79] Nakagawa J., Ishikura S., Asami J., Isaji T., Usami N., Hara A., Sakurai T., Tsuritani K., Oda K., Takahashi M. (2002). Molecular characterization of mammalian dicarbonyl/L-xylulose reductase and its localization in kidney. J. Biol. Chem..

[80] Lane A.B., Jenkins T. (1985). Human L-xylulose reductase variation: Family and population studies. Ann. Hum. Genet..

[81] Barovic M., Hahn J.J., Heinrich A., Adhikari T., Schwarz P., Mirtschink P., Funk A., Kabisch S., Pfeiffer A.F.H., Blüher M. (2025). Proteomic and metabolomic signatures in prediabetes progressing to diabetes or reversing to normoglycemia within 1 Year. Diabetes Care.

[82] Depintor T.S., Freitas F.C.P., Hernandes N., Nunes F.M.F., Simões Z.L.P. (2025). Interactions of juvenile hormone, 20-hydroxyecdysone, developmental genes, and miRNAs during pupal development in Apis mellifera. Sci. Rep..

[83] Shalem Y., Goldberg T.S., Bloch G. (2025). Juvenile hormone signaling and social complexity in the Hymenoptera. Curr. Opin. Insect Sci..

[84] Shpigler H.Y., Herb B., Drnevich J., Band M., Robinson G.E., Bloch G. (2020). Juvenile hormone regulates brain-reproduction tradeoff in bumble bees but not in honey bees. Horm. Behav..

[85] Corona M., Velarde R.A., Remolina S., Moran-Lauter A., Wang Y., Hughes K.A., Robinson G.E. (2007). Vitellogenin, juvenile hormone, insulin signaling, and queen honey bee longevity. Proc. Natl. Acad. Sci. USA.

[86] Gong Z.X., Cheng F.P., Xu J.N., Yan W.Y., Wang Z.L. (2023). The Juvenile-Hormone-Responsive factor AmKr-h1 regulates caste differentiation in honey bees. Biomolecules.

[87] Bomtorin A.D., Mackert A., Rosa G.C., Moda L.M., Martins J.R., Bitondi M.M., Hartfelder K., Simões Z.L. (2014). Juvenile hormone biosynthesis gene expression in the corpora allata of honey bee *(Apis mellifera* L.) female castes. PLoS ONE.

[88] Zhang Z., Liu X., Shiotsuki T., Wang Z., Xu X., Huang Y., Li M., Li K., Tan A. (2017). Depletion of juvenile hormone esterase extends larval growth in *Bombyx mori*. Insect Biochem. Mol. Biol..

[89] Tskhovrebova L., Trinick J. (2003). Titin: Properties and family relationships. Nat. Rev. Mol. Cell Biol..

[90] Qu X., Lu H., Zhang X., Xin L., Chen X. (2026). TITIN Regulates Body Size via the JH–JHE Pathway in Honeybees (*Apis mellifera*). Int. J. Mol. Sci..

[91] Bonev B., Cavalli G. (2016). Organization and function of the 3D genome. Nat. Rev. Genet..

[92] Busslinger G.A., Stocsits R.R., van der Lelij P., Axelsson E., Tedeschi A., Galjart N., Peters J.-M. (2017). Cohesin is positioned in mammalian genomes by transcription, CTCF and Wapl. Nature.

[93] Zuin J., Dixon J.R., van der Reijden M.I., Ye Z., Kolovos P., Brouwer R.W., van de Corput M.P., van de Werken H.J., Knoch T.A., van Ijcken W.F. (2014). Cohesin and CTCF differentially affect chromatin architecture and gene expression in human cells. Proc. Natl. Acad. Sci. USA.

[94] Narendra V., Rocha P.P., An D., Raviram R., Skok J.A., Mazzoni E.O., Reinberg D. (2015). CTCF establishes discrete functional chromatin domains at the *Hox* clusters during differentiation. Science.

[95] Seitan V.C., Faure A.J., Zhan Y., McCord R.P., Lajoie B.R., Ing-Simmons E., Lenhard B., Giorgetti L., Heard E., Fisher A.G. (2013). Cohesin-based chromatin interactions enable regulated gene expression within preexisting architectural compartments. Genome Res..

[96] Sofueva S., Yaffe E., Chan W.C., Georgopoulou D., Vietri Rudan M., Mira-Bontenbal H., Pollard S.M., Schroth G.P., Tanay A., Hadjur S. (2013). Cohesin-mediated interactions organize chromosomal domain architecture. EMBO J..

[97] Arzate-Mejía R.G., Josué Cerecedo-Castillo A., Guerrero G., Furlan-Magaril M., Recillas-Targa F. (2020). In situ dissection of domain boundaries affect genome topology and gene transcription in *Drosophila*. Nat. Commun..

[98] Sexton T., Yaffe E., Kenigsberg E., Bantignies F., Leblanc B., Hoichman M., Parrinello H., Tanay A., Cavalli G. (2012). Three-dimensional folding and functional organization principles of the *Drosophila* genome. Cell.

