# Supplementary material for: Genome-Wide Chromatin Structure Changes During Caste Differentiation in Honeybees
Source: Cells. 2026 Oct 19;15(20):1899. doi: 10.3390/cells15201899 (PMC13702978; doi:10.3390/cells15201899)
Supplement: Supplementary file 1 [file cells-15-01899-s001.zip › Supplementary Figure/20260826-Supplementary Figure-Cells.pdf]

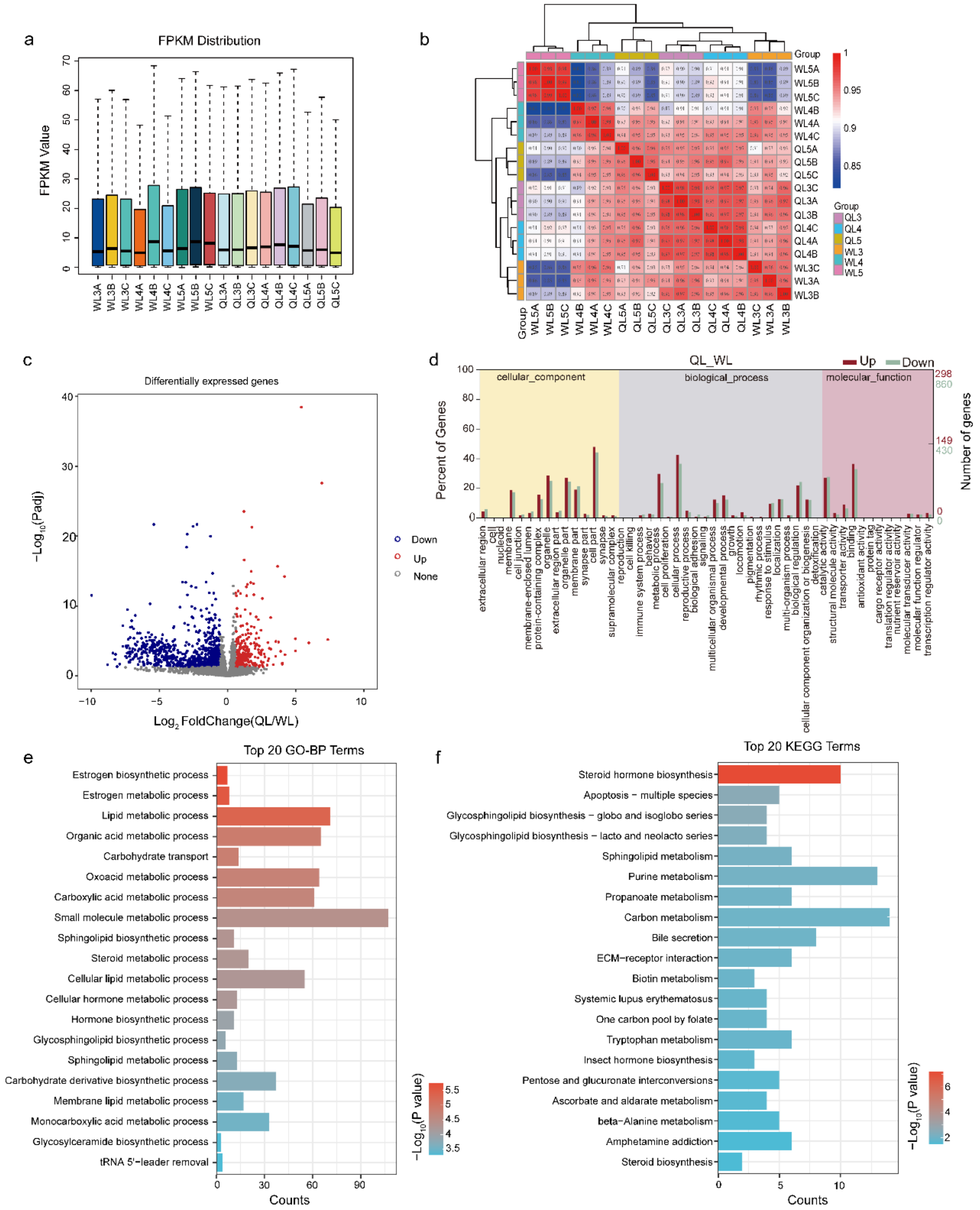

Figure S1. Specific expression genes of QL and WL in developmental stage. (**a**) The box plot of FPKM value in QL and WL; (**b**) Heatmap shows the cluster classification for all RNA-seq libraries according to their normalized FPKM value; (**c**) Volcano plot showing DEGs form comparison of QL and WL; Number in red indicates up-regulated and number in blue indicates down-regulated. (**d**) Overrepresented GO terms enriched with DEGs between QL and WL; (**e-f**) Enriched GO-BP terms and KEGG pathways with DEGs between QL and WL, shown are top 20 terms.

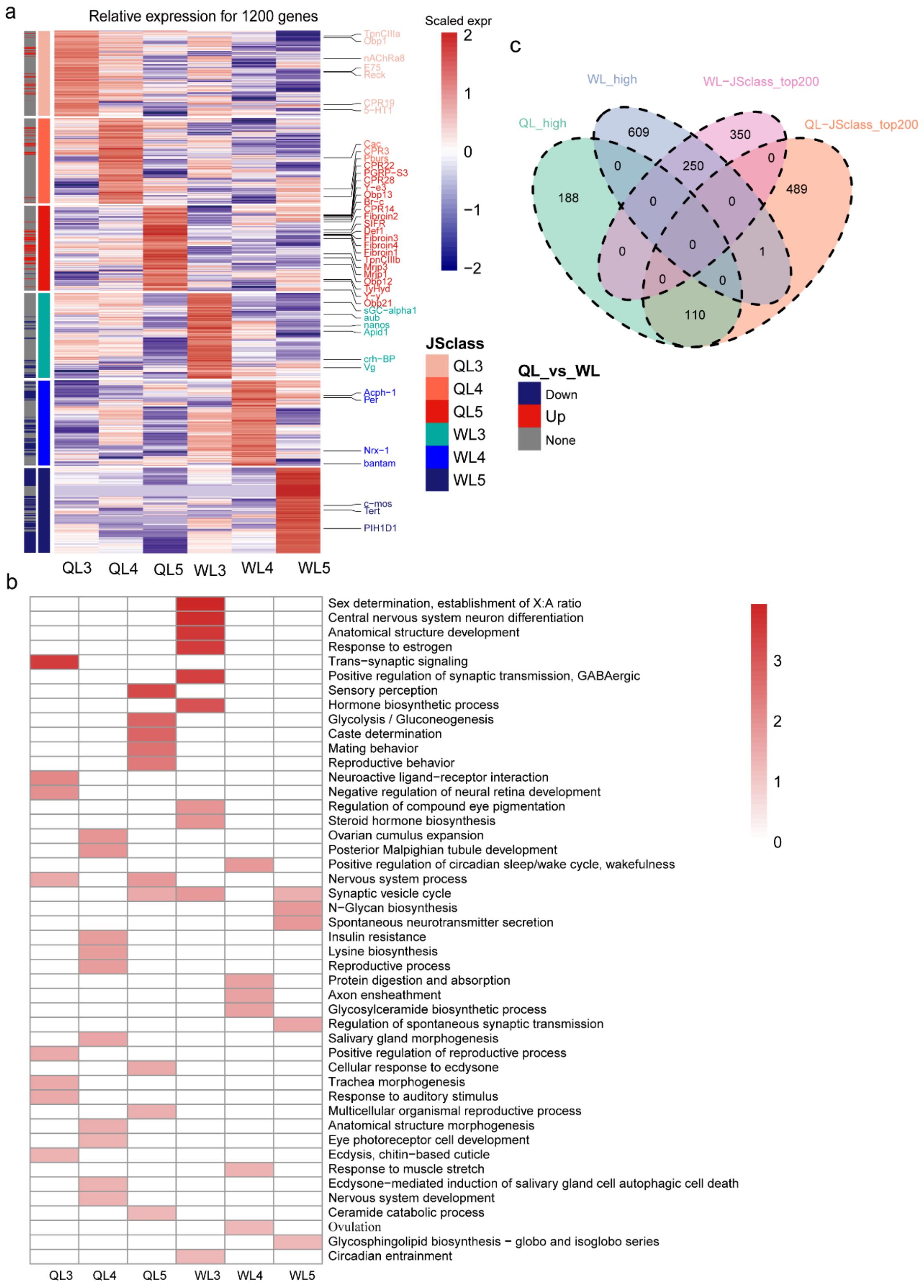

Figure S2. Stage-specific expression genes of QL and WL in developmental stage. (**a**) Heatmap shows the stage-specific genes across all stages. Shown are tissue specific genes with JS scores > 0.3; (**b**) Heatmap shows the represented enriched biological processes with stage-specific genes; (**c**) The venn diagram shows the number of top 200 high expressed DEGs and JSclass_top200 stage-specific genes in QL and WL.

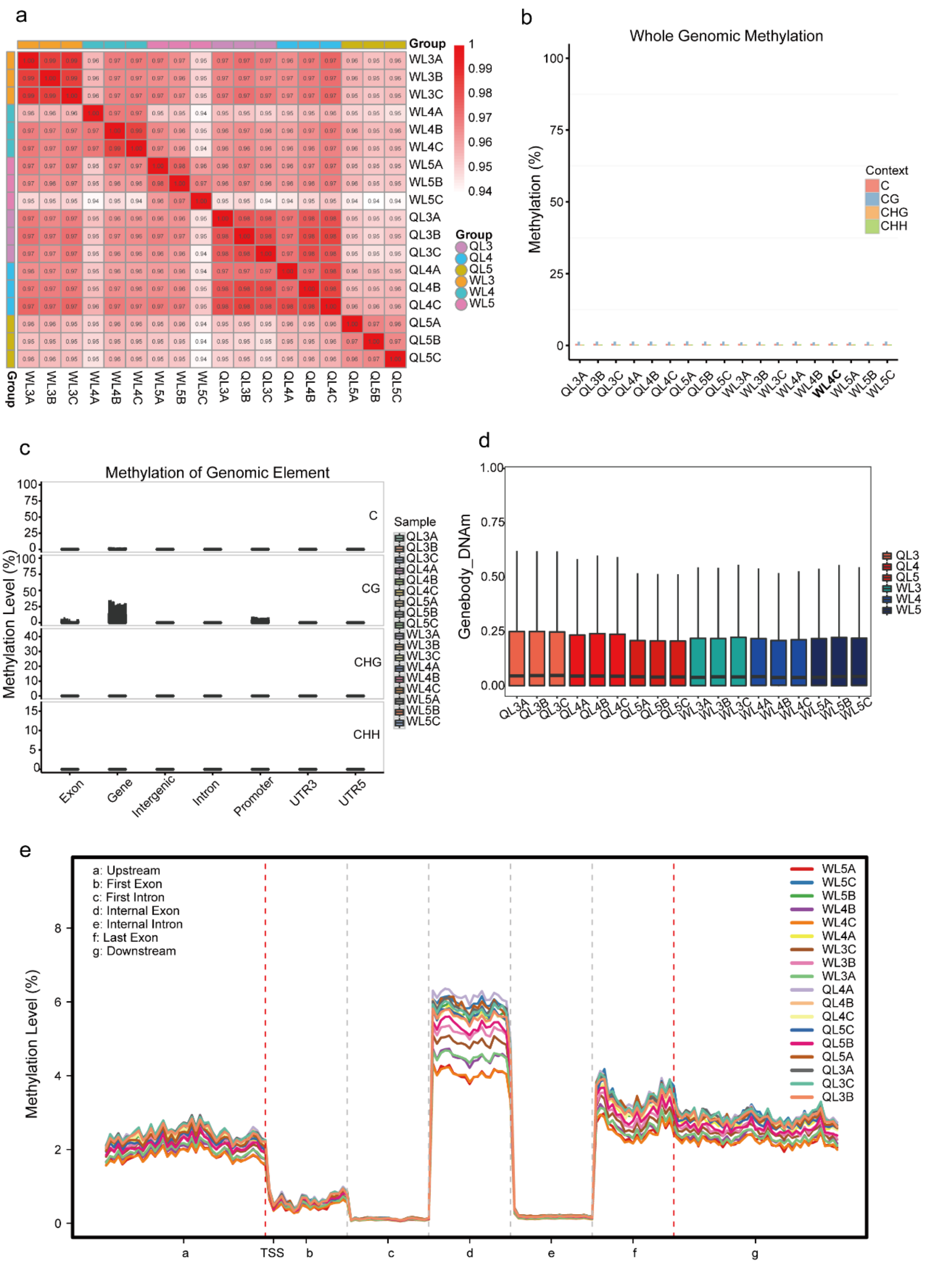

Figure S3. DNA methylation landscape in several developmental stage. (**a**) Heatmap shows the cluster classification for all BS-seq libraries according to all gene body DNAm; (**b**) Boxplot of methyl cytosines in the four contexts identified in QL and WL; (**c**) Distribution of methylated cytosines in the specific genomic elements in QL and WL; (**d**) Boxplot of gene body DNAm in QL and WL; (**e**)Distribution of methylation levels in the specific gene function regions.

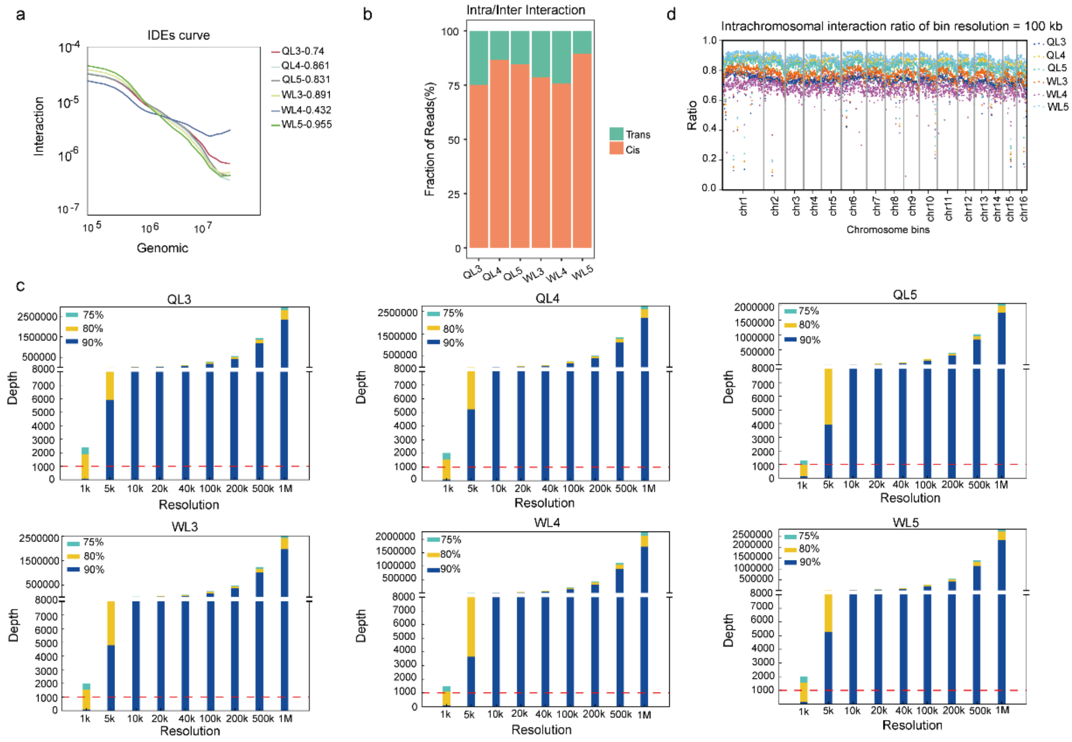

Figure S4. The characteristics of Hi-C data set in QL and WL. (**a**) The IDEs curve across all different stages; (**b**) The total intra- and inter chromosome interactions across all different stages; (**c**) Resolution depth across all different stages; (d) Intrachromosomal interaction ratio at 100 kb resolution.

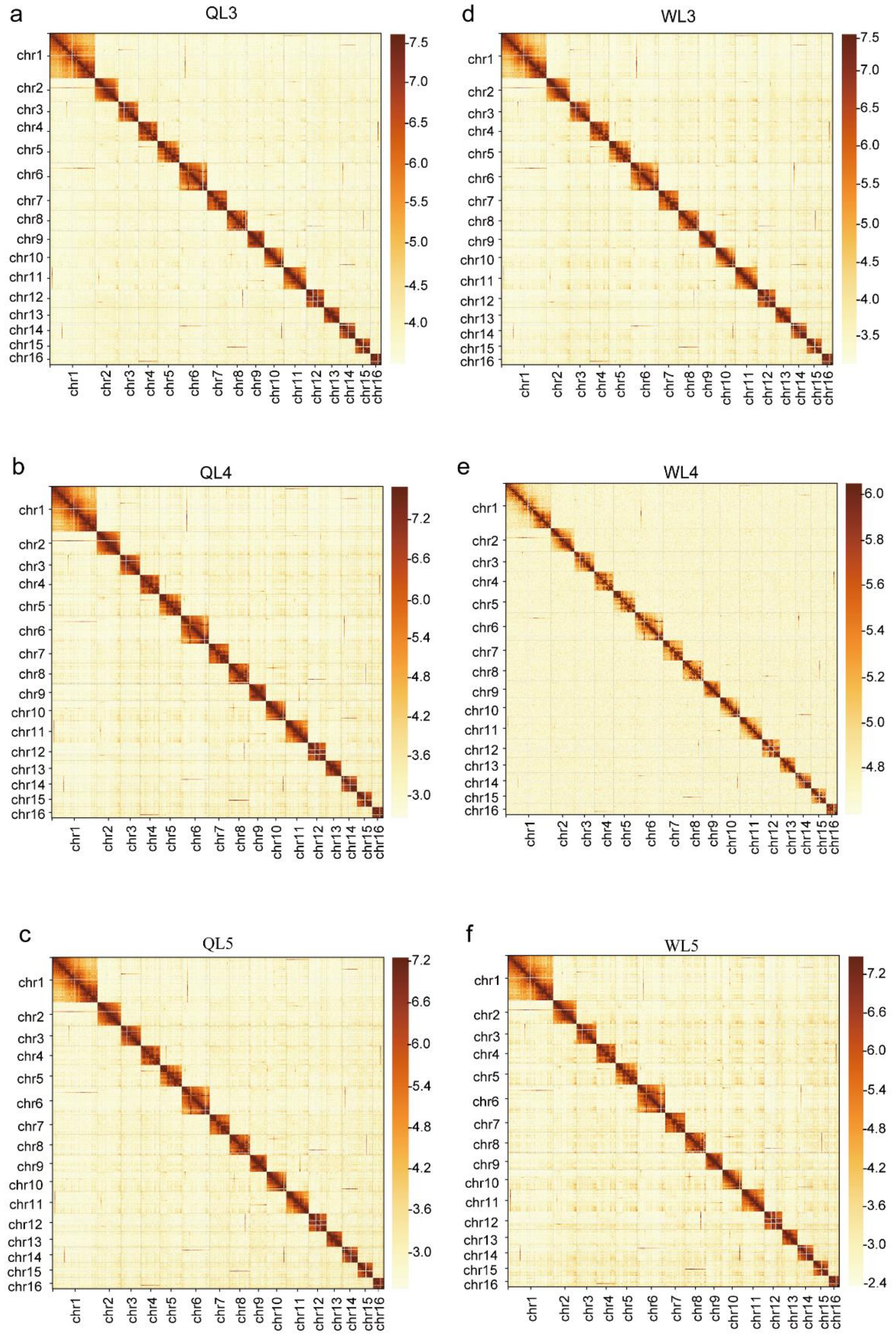

Figure S5. The whole genome interactions in QL and WL. (**a-c**) Normalized Hi-C interaction heatmaps at 100 kb resolution across QL; (d-f) Normalized Hi-C interaction heatmaps at 100 kb resolution across WL.

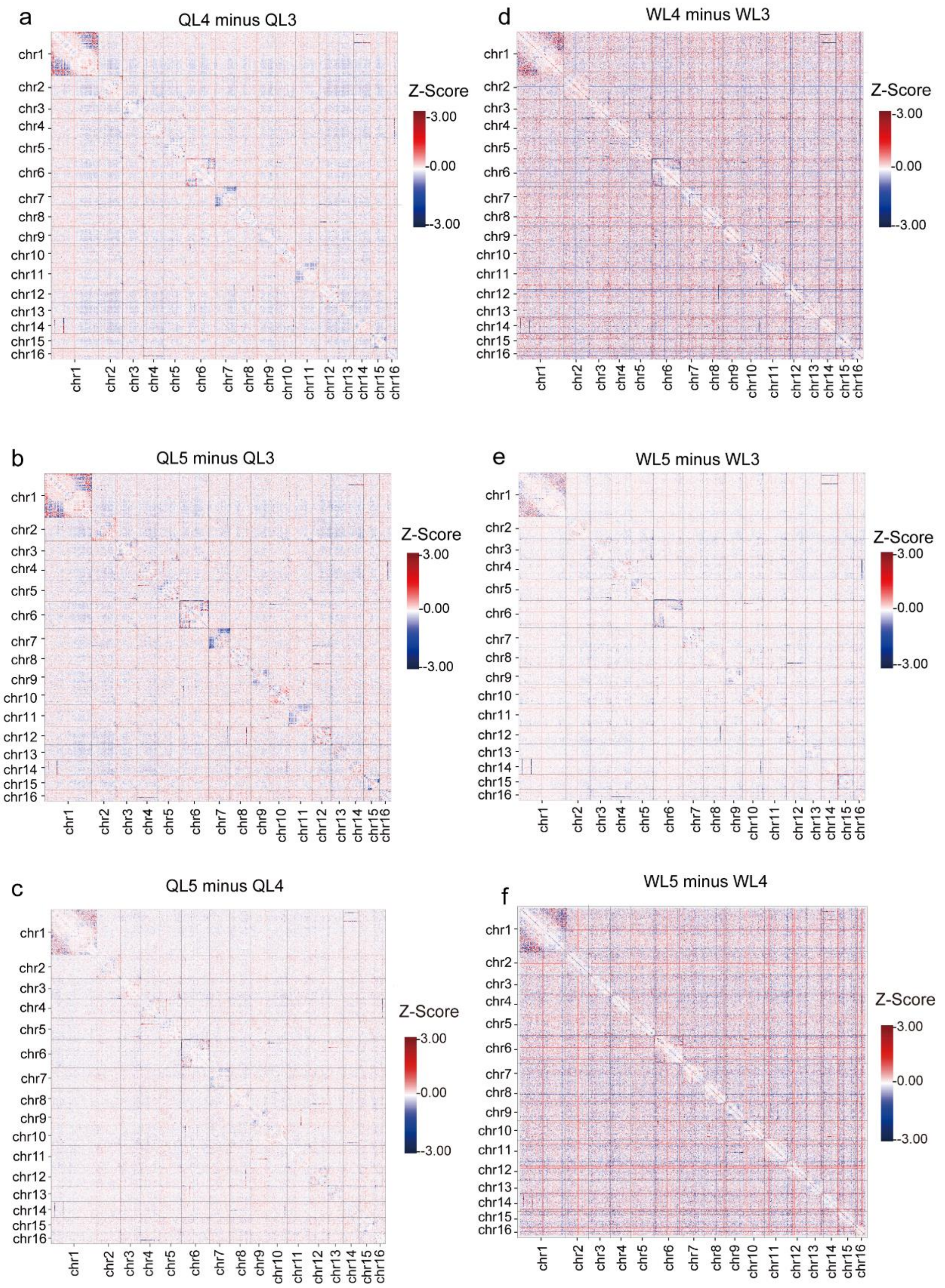

Figure S6. The differential whole genome interactions between QL and WL. (**a-c**) he differential whole genome interactions between QL; (**d-f**) The differential whole genome interactions between WL.

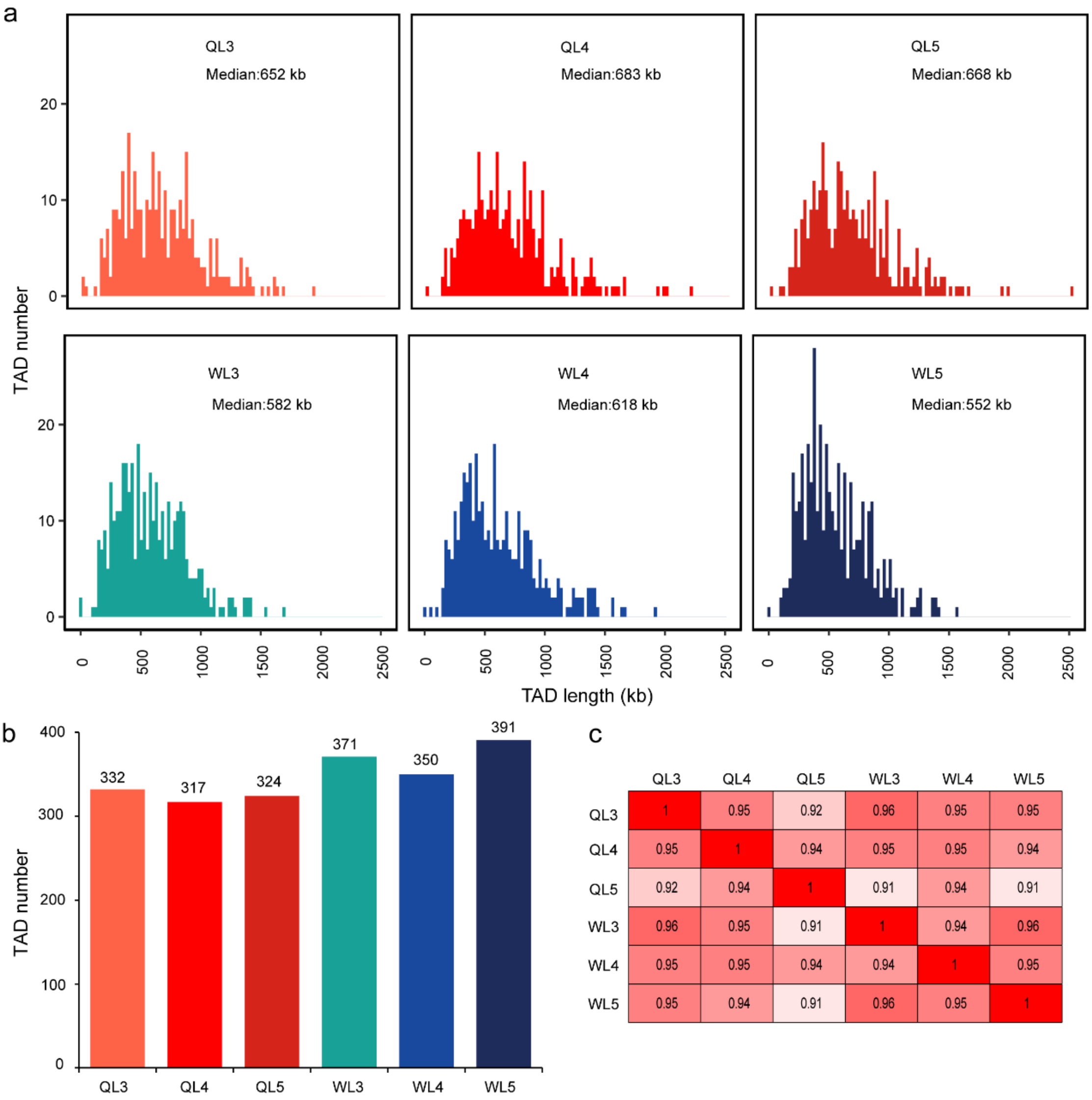

Figure S7. Characteristics of TADs in QL and WL. (**a**) Frequency distribution histograms of TAD length in QL and WL; (**b**) TAD number in QL and WL; (**c**) Spearman's r heatmap of the directionality index (DI) among the six samples, showing a high reproducibility within biological replicates (Spearman's r > 0.91).

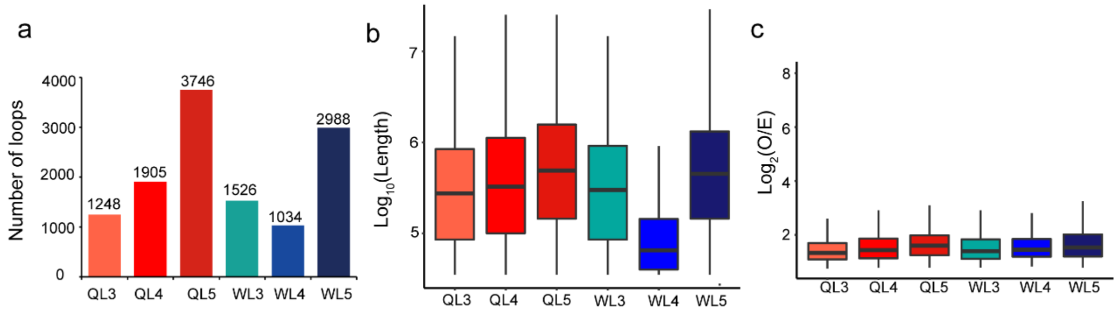

Figure S8. Characteristics of TADs in QL and WL. (**a**) Number of loops in QL and WL; (b) Length (Log2(Length)) of loops in QL and WL; In the boxplot, the internal line indicates the median, the box limits indicate the upper and lower quartiles and the whiskers extend to 1.5 IQR from the quartiles. (**c**) Observation/Expectation value (Log2(O/E)) of loops in QL and WL.
